# The role of introgression and ecotypic parallelism in delineating intraspecific conservation units

**DOI:** 10.1111/mec.15522

**Published:** 2020-07-11

**Authors:** Rebecca S. Taylor, Micheline Manseau, Rebekah L. Horn, Sonesinh Keobouasone, G. Brian Golding, Paul J. Wilson

**Affiliations:** ^1^ Biology Department Trent University Peterborough ON Canada; ^2^ Landscape Science and Technology Division Environment and Climate Change Canada Ottawa ON Canada; ^3^ Department of Biology McMaster University Hamilton ON Canada; ^4^ Columbia River Inter‐Tribal Fish Commission Hagerman ID USA

**Keywords:** adaptive introgression, conservation legislation, demographic history, introgressed populations, management units, parallel evolution

## Abstract

Parallel evolution can occur through selection on novel mutations, standing genetic variation or adaptive introgression. Uncovering parallelism and introgressed populations can complicate management of threatened species as parallelism may have influenced conservation unit designations and admixed populations are not generally considered under legislations. We examined high coverage whole‐genome sequences of 30 caribou (*Rangifer tarandus*) from across North America and Greenland, representing divergent intraspecific lineages, to investigate parallelism and levels of introgression contributing to the formation of ecotypes. Caribou are split into four subspecies and 11 extant conservation units, known as designatable units (DUs), in Canada. Using genomes from all four subspecies and six DUs, we undertake demographic reconstruction and confirm two previously inferred instances of parallel evolution in the woodland subspecies and uncover an additional instance of parallelism of the eastern migratory ecotype. Detailed investigations reveal introgression in the woodland subspecies, with introgressed regions found spread throughout the genomes encompassing both neutral and functional sites. Our investigations using whole genomes highlight the difficulties in unequivocally demonstrating parallelism through adaptive introgression in nonmodel species with complex demographic histories, with standing variation and introgression both potentially involved. Additionally, the impact of parallelism and introgression on conservation policy for management units needs to be considered in general, and the caribou designations will need amending in light of our results. Uncovering and decoupling parallelism and differential patterns of introgression will become prevalent with the availability of comprehensive genomic data from nonmodel species, and we highlight the need to incorporate this into conservation unit designations.

## 1 INTRODUCTION

Parallel evolution is a process where divergent populations living in similar environments evolve the same or similar traits (Lamichhaney et al., 2017; Oke, Rolshausen, Leblond, & Hendry, 2017). Cases of parallelism can occur by selection on either new mutations, standing genetic variation or adaptive introgression, whereby adaptive genetic variation is transmitted by interbreeding into a new population or species (Fraser & Whiting, 2019; Hedrick, 2013; Lee & Coop, 2017; Macpherson & Nuismer, 2017). Adaptive introgression between divergent lineages can facilitate parallel evolution even if traits are controlled by more than one locus, and could therefore be difficult to distinguish from parallel evolution from standing genetic variation (Bassham, Catchen, Lescak, von Hippel, & Cresko, 2018; Fraser & Whiting, 2019; Hedrick, 2013; Lee & Coop, 2017). Parallel evolution through adaptive introgression and selection on standing variation may also happen in concert (Bassham et al., 2018; Fraser & Whiting, 2019; Lee & Coop, 2017).

Recent findings show high levels of introgression among taxa (Kumar et al., 2017; Mallet, 2005; Taylor & Larson, 2019) and highly selective introgression of important adaptive genomic regions (e.g. Poelstra et al., 2014; Song et al., 2011; The Heliconius Genome Consortium, 2012). However, admixed or hybrid populations, populations which have resulted in different genetic lineages interbreeding, are not generally considered under current conservation legislations (Fitzpatrick, Ryan, Johnson, Corush, & Carter, 2015; Rius & Darling, 2014; vonHoldt, Brzeski, Wilcove, & Rutledge, 2018) and when discussed, the focus is typically on interspecies hybrids and not conservation units below the species level (Fitzpatrick et al., 2015). Given the current extinction crisis under climate change also resulting in range shifts and increased secondary contact and therefore admixture (Garroway et al., 2010; Gómez, González‐Megías, Lorite, Abdelaziz, & Perfectti, 2015), new management frameworks will be required to encompass more complex evolutionary histories (vonHoldt et al., 2018).

**FIGURE 1:**
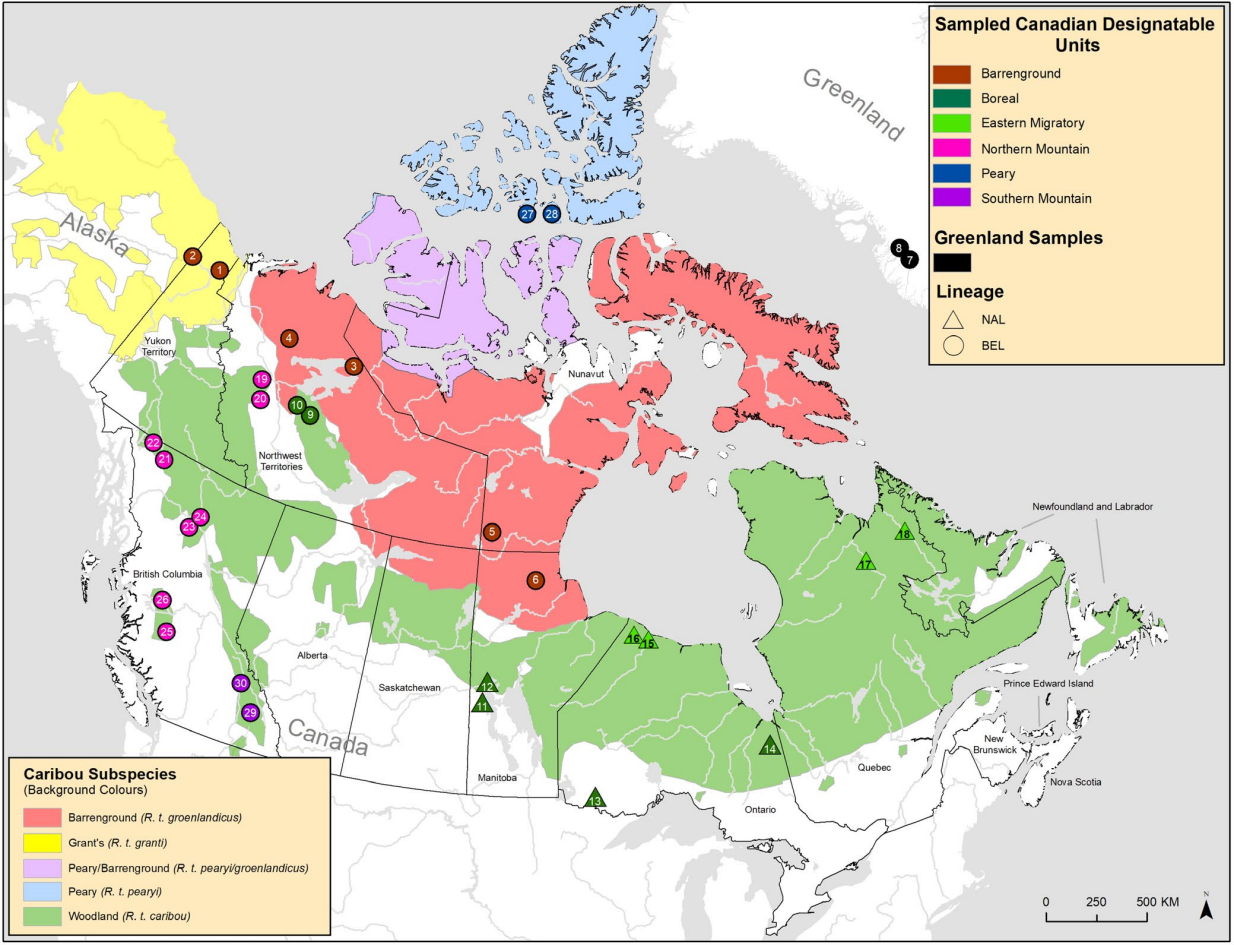
Range of caribou in North America. Background colours show the ranges of the four subspecies (*R. t. caribou; R. t. groenlandicus; R. t. pearyi; R. t. granti*). Circles and triangles indicate sampling locations for this study and are coloured by designatable unit. We also included two genomes from Greenland shown by the black circles. A circle indicates that the sample is from the BEL mitochondrial lineage and a triangle means the sample is from the NAL mitochondrial lineage. Sample numbers 1 and 2 are from the Yukon Porcupine herd, 3 and 4 from the Northwest Territories Bluenose herd, 5 and 6 from the Manitoba Qamanirijuaq herd, 7 and 8 from Western Greenland Kangerlussuaq, 9 and 10 from the Northwest Territories Sahtú region, 11 and 12 from the Manitoba Naosap herd, 13 from Ontario Ignace 14 from Ontario Cochrane, 15 and 16 from the Ontario Pen Island herd, 17 and 18 from the Quebec George River herd, 19 and 20 from the Northwest Territories, Redstone herd, 21 and 22 from the British Columbia Atlin herd, 23 and 24 from the British Columbia Frog herd, 25 and 26 from the British Columbia Itcha‐Ilgachuz herd, 27 and 28 from Nunavut Bathurst Island and 29 and 30 from the British Columbia Columbia North herd[Colour figure can be viewed at wileyonlinelibrary.com]

Here, we investigate nuclear genomic structure of caribou (*Rangifer tarandus*) across North America and Greenland and investigate how intraspecific parallelism contributed to the formation of caribou ecotypes. We then investigate levels of introgression between divergent intraspecific lineages. In Canada, there are four caribou subspecies largely based on morphology (Banfield, 1961; Figure 1). They are distributed in widely different ecozones, including the High Arctic, mountains, taiga and boreal forests (Banfield, 1961; COSEWIC, 2011). They display evidence of local adaptation, with differences in morphology, diet, behaviour and life history in different regions, leading to the classification of 12 designatable units (DUs; 11 extant and 1 extinct; COSEWIC, 2011; Figure S1), often referred to as ecotypes, by the Committee on the Status of Endangered Wildlife in Canada (COSEWIC, 2011). Importantly, all 11 extant ecotypes are now listed as at risk of extinction (COSEWIC, 2011, 2014a, 2014b, 2015a, 2015b, 2016, 2017a, 2017b) and many have been declining rapidly due to human‐mediated disturbances including climate change (Festa‐Bianchet, Ray, Boutin, Côté, & Gunn, 2011; Vors & Boyce, 2009; Weckworth, Hebblewhite, Mariani, & Musiani, 2018). Additionally, caribou are of huge cultural, spiritual and economic significance to many indigenous communities (Festa‐Bianchet et al., 2011; Polfus et al., 2016). It is also a keystone species for the ecosystem, important for vegetation structure, nitrogen cycling and predator populations (Festa‐Bianchet et al., 2011).

Previous mitochondrial DNA studies indicate two major phylogenetic lineages of caribou in North America which likely have origins in different glacial refugia (Cronin, MacNeil, & Patton, 2005; Flagstad & Røed, 2003; Klütsch, Manseau, & Wilson, 2012; Weckworth, Musiani, Devitt, Hebblewhite, & Mariani, 2012). The mitochondrial lineages do not line up with the current subspecies or DU designations in all areas. For example, the boreal DU, which extends from the east coast of Canada to the northern regions of the Northwest Territories, contains individuals belonging to the North American phylogenetic lineage, or NAL, in the central and eastern part of the range (Klütsch et al., 2012; Polfus, Manseau, Klütsch, Simmons, & Wilson, 2017). However, boreal caribou from the northern part of the Northwest Territories belong to the Beringian–Eurasian phylogenetic lineage, or BEL, indicating potential parallel evolution (Polfus et al., 2017). The northern mountain DU also sits within the BEL, even though they belong to the woodland subspecies along with the boreal DU (Polfus et al., 2017; Figure 1) also indicating potential parallel evolution. Additionally, the eastern migratory DU has two disjunct ranges, one in northern Manitoba and Ontario and the other in northern Quebec and Labrador (Figure S1). Eastern migratory caribou from the Ontario and Manitoba region were found to be an admixture of boreal caribou from the NAL lineage and barrenground caribou from the BEL lineage (Klütsch, Manseau, Trim, Polfus, & Wilson, 2016). However, it is unknown whether the Quebec and Labrador eastern migratory ecotype share the same origin.

We examined high coverage whole‐genome sequences of 30 caribou in the most comprehensive study to date covering six DUs and all four subspecies (Figure 1; Table 1). We used genomewide variation using population and phylogenomic approaches to investigate (a) what is the nuclear phylogenomic structure of caribou across North America and Greenland and does this match the mitochondrial lineages and potential origins in two major refugia? (b) Do we confirm parallel evolution of northern mountain and boreal caribou in the northern part of the range with the rest of the woodland subspecies and did the eastern migratory caribou evolve the same phenotype in parallel in the two disjunct ranges? (c) Was there introgression among the caribou lineages we uncovered using phylogenomic analyses? What do these patterns look like across the genome and do we see introgression of genes putatively suggesting adaptive introgression in facilitating the parallel evolution of ecotypes? And finally, (d) are the eastern migratory caribou from Quebec/Labrador admixed as was found for eastern migratory caribou from Ontario/Manitoba? Issues of parallelism and complex patterns of introgression will certainly become more prevalent with advances in sequencing technologies and we discuss how the definition and delineation of conservation units could be informed by our results.

**TABLE 1:** Sample information (location and reference numbers), subspecies, designatable unit (DU) and mitochondrial lineage for each sampled caribou

Location and reference numbers	Subspecies	DU	Mitochondrial Lineage	Figure 1 Reference
Yukon Porcupine herd 27,737 and 27,738	Grant's caribou (*R. t. grantii*)	Barrenground	BEL	1, 2
Northwest Territories Bluenose herd 27,177 and 27,186	Barrenground (*R. t. groenlandicus*)	Barrenground	BEL	3, 4
Manitoba Qamanirijuaq herd 21,332 and 21,350	Barrenground (*R. t. groenlandicus*)	Barrenground	BEL	5, 6
Western Greenland Kangerlussuaq 41,660 and 41,667	Barrenground (*R. t. groenlandicus*)	n/a	BEL	7, 8
Northwest Territories Sahtú region 17,825 and 35,082	Woodland (*R. t. caribou*)	Boreal	BEL	9, 10
Manitoba (The Pas) Naosap herd 35,324	Woodland (*R. t. caribou*)	Boreal	NAL	11
Manitoba (Snow Lake) Naosap herd 35,326	Woodland (*R. t. caribou*)	Boreal	NAL	12
Ontario Ignace 39,590	Woodland (*R. t. caribou*)	Boreal	NAL	13
Ontario Cochrane 39,654	Woodland (*R. t. caribou*)	Boreal	NAL	14
Ontario Pen Island herd 20,917 and 34,590	Woodland (*R. t. caribou*)	Eastern migratory	NAL	15, 16
Quebec George River herd 27,689 and 27,694	Woodland (*R. t. caribou*)	Eastern migratory	NAL	17, 18
Northwest Territories Redstone herd 15,460 and 17,896	Woodland (*R. t. caribou*)	Northern mountain	BEL	19, 20
British Columbia Atlin herd 28,575 and 28,580	Woodland (*R. t. caribou*)	Northern mountain	BEL	21, 22
British Columbia Frog herd 28,327 and 28,337	Woodland (*R. t. caribou*)	Northern mountain	BEL	23, 24
British Columbia Itcha‐Ilgachuz herd 28,395 and 28,402	Woodland (*R. t. caribou*)	Northern mountain	BEL	25, 26
Nunavut Bathurst Island 34,549 and 34,550	Peary (*R. t. pearyi*)	Peary	BEL	27, 28
British Columbia Columbia North herd 28,646 and 28,649	Woodland (*R. t. caribou*)	Southern mountain	BEL	29, 30
China Greater Khingan Mountains, Inner Mongolia Autonomous Region	Domesticated Reindeer (See Li et al. 2017)	n/a	BEL	n/a

## 2 MATERIALS AND METHODS

### 2.1 Sample collection, extraction and sequencing

Tissue was collected from 28 caribou from across Canada and two caribou from Greenland between 1992 and 2015, representing all four subspecies and six Canadian designatable units (DUs; Figure 1, Table 1 and Table S1). Samples were collected on road kills or from harvested animals by biologists or veterinarians with the British Columbia, Manitoba and Ontario provincial governments, the Canadian federal government, the Greenland government, the Sahtú Renewable Resources Board, The Royal Ontario Museum, the University of Manitoba and an independent consultant (see Table S1). Tissues were stored in RNA later ICE (Thermo Fisher Scientific, MA, USA). Phenol chloroform extractions were performed on three of the samples (The Pas, Snow Lake and Ignace) using 0.2 g of tissue, and eluted in Tris‐ethylenediaminetetraacetic acid (TE) buffer at 100 µl. The other samples were extracted using a Qiagen DNAeasy tissue extraction kit following the manufacturer's instructions (Qiagen, Hilden, Germany). The samples were run on a Qubit fluorometer (Thermo Fisher Scientific, MA, USA) using the High Sensitivity Assay Kit and normalized to 20ng/µl at a final volume of 50 µl. The DNA was shipped to The Centre for Applied Genomics (TCAG) at the Hospital for Sick Children (Toronto, Ontario) for library preparation and sequencing. The samples were each run on one lane of an Illumina HiSeq X (Illumina, San Diego, CA, USA), for a total of 30 lanes of sequencing. All raw reads are available at the National Centre for Biotechnology (NCBI) under the BioProject Accession no. PRJNA634908.

### 2.2 Filtering raw reads and variant calling

We used Trimgalore 0.4.2 (available here: https://github.com/FelixKrueger/TrimGalore), a wrapper script for CutAdapt (Martin, 2011), to remove sequencing adaptors and to trim low‐quality ends from reads with a phred quality score below 30. Reads were aligned to the caribou reference genome (Taylor et al., 2019) using Bowtie2 2.3.0 (Langmead & Salzberg, 2012), and the SAM file converted to a BAM file using Samtools 1.5 (Li et al., 2009). We removed duplicate reads and added correct read group information to each BAM file using Picard 2.17.3 (Available: http://broadinstitute.github.io/picard/). We then sorted the BAM file using Samtools 1.5 and built an index using Picard. All BAM files were checked using FastQC 0.11.8 (Andrews, 2010), and we calculated the mean depth of coverage for each BAM file using Samtools.

In addition to the 30 caribou genomes we sequenced, we also used a reindeer genome from a domesticated animal from Inner Mongolia, sequenced by Li et al., (2017). The reads were downloaded from NCBI (SRR5763125‐SRR5763133) and mapped back to the caribou reference genome using the same methods as above. After using FastQC, because adaptor contamination was detected, as well as duplicate reads, we used ClipReads in GatK to remove Nextera adaptor contamination and reremoved duplicates using Picard. We then rechecked the file using FastQC and found we had successfully removed the contamination. Some sequence duplication was still detected, however, with the per cent of sequences remaining if deduplicated at 55.62% according to the FastQC report, and so results from the reindeer sequence may need to be treated with caution.

We ran each BAM file through BUSCO (Benchmarking Universal Single‐Copy Orthologs 3.0.2; Waterhouse et al., 2018) to reconstruct 4,104 conserved mammalian genes to assess the completeness of each genome. As our reference genome reconstructed 3,820 (93.1%; Taylor et al., 2019) complete mammalian BUSCO genes, this represents an upper limit for our resequenced individuals. We used Haplotype Caller in gatk 3.8 (McKenna et al., 2010) to call variants and produce a variant call format (VCF) file for each caribou. Individual VCF files were combined using the Combine GVCFs function, and then, we performed joint genotyping using Genotype GVCFs, both in GATK, to produce a VCF file with all caribou and the reindeer. For some PCA's (see below), we also made VCF files containing subsets of individuals by rerunning the Combine GVCFs and Genotype GVCFs functions with only the individuals needed, which were also filtered as below.

We downloaded the raw reads for a Sitka deer (*Odocoileus hemionus sitkensis*) genome from the NCBI database (Bioproject PRJNA476345, run SRR7407804) sequenced as part of the CanSeq150 Initiative, to use as an outgroup. We aligned and filtered the reads in the same way as for the caribou genomes to produce an individual VCF file. We then used the Combine GVCFs function and performed joint genotyping using Genotype GVCFs, both in GATK, to produce a VCF file with all caribou, the reindeer and the Sitka deer, for analyses requiring an outgroup.

We did some additional filtering on the combined VCF files to ensure quality. We used VCFtools 0.1.14 (Danecek et al., 2011) to do two rounds of filtering. First, we removed indels (using the remove‐indels command), and any site with a depth of less than 10 or more than 80 (approximately double the average depth across the genome, using the min‐meanDP and max‐meanDP commands) and removed any low‐quality genotype calls, with a score below 20, (using the minGQ command) which in VCFtools are changed to missing data. In the second round, we filtered to remove genotypes with more than 10% missing data (using the max‐missing command). We did not filter to remove any SNP with a minor allele frequency (MAF) as we have only one or two individuals from each location and this results in removing the private sites, instead relying on very high depth and stringent filtering to ensure a high‐quality data set. However, we did conduct the PCA removing sites with an MAF of less than 0.05 and these looked identical to the data without the MAF filter (Figures S2–S5). The combined VCF file used for analyses with all individuals apart from the Sitka deer contained 34,573,476 SNPs, and the VCF including the Sitka deer contained 65,412,957 SNPs. After filtering, we measured the mean depth (using the depth command), the frequency of missing data (using the missing‐indv command) and the inbreeding coefficient, *F* (using the het command), for each individual in the final VCF file of 30 caribou plus the reindeer using VCFtools.

### 2.3 Population and phylogenomic structure

We performed principal component analyses (PCA) in r 3.4.4 (R Core Team, 2018) using the packages vcfR (Knaus & Grüwald, 2017) and Adegenet (Jombart, 2008). The PCA was done on the VCF file containing all caribou and the reindeer (but not the Sitka deer). We then ran subsets of individuals on different PCAs to gain higher resolution of different lineages (see Results).

We used VCFkit (available here: https://vcf‐kit.readthedocs.io/en/latest/, using numpy 1.14 as the programme does not work with newer versions) to generate a fasta file using the ‘phylo fasta’ command. The programme concatenates SNPs for each sample, using the first genotype of each allele (e.g. for diploids where the genotype is A/T, the A is used) and replacing missing values with an N. We ran this on the VCF file without the Sitka deer to create an unrooted tree as including the Sitka deer pushed all caribou too closely together to discern the branches. The resulting file was input into RAxML 8 (Stamatakis, 2014) and run using the GTRGAMMA model and 1,000 bootstrap replicates. We visualized the best tree in FigTree 1.4.2 (https://github.com/rambaut/figtree). We also aligned each of the conserved mammalian genes extracted from the genomes using BUSCO (above) to construct phylogenies, from which we made a consensus tree. We used the Sitka deer outgroup to root the tree. We used muscle (Edgar, 2004) to align the sequences for each individual to create a combined fasta file for each gene. We then used RAxML as above to create a gene tree for each file and then used ASTRAL‐III (Zhang, Rabiee, Sayyari, & Mirarab, 2018) to create a consensus tree which was visualized in FigTree.

We used the populations module in Stacks 2.4.1 (Catchen, Hohenlohe, Bassham, Amores, & Cresko, 2013) to convert our VCF files (both with and without the Sitka deer) into an input file for Treemix 1.13 (Pickrell & Pritchard, 2012). We ran Treemix from 0 to 9 migration events, with ten iterations of each, grouping the SNPs in windows to account for possible linkage using a block size of 1,000 SNPs for seven of the iterations and 5,000 SNPs for three of the iterations (because the OptM package, below, must have different likelihood scores between iterations). We plotted the resulting trees, and the residual plots, in RStudio 1.0.136 (RStudio Team, 2015). We then used the R package OptM (available here: https://cran.r-project.org/web/packages/OptM/index.html) to calculate the second‐order rate of change in the log‐likelihood of the different migration events (the ad hoc statistic delta M) to help infer how many migration events to visualize.

### 2.4 Demographic reconstruction and admixture analyses

We made a consensus fastq file for each caribou and the reindeer from their BAM files, using the Samtools and BCFtools 1.5. This was converted into an input file using the ‘fq2psmcfa’ command and run using the ‘psmc’ command in Pairwise Sequentially Markovian Coalescent (PSMC) model in PSMC (Li & Durbin, 2011) to investigate past effective population size changes. These were plotted using the general mammal mutation rate of 1.0E‐9 per year (Li & Durbin, 2011) and a generation time of 7 years (COSEWIC, 2011, 2014a, 2014b, 2015a, 2015b, 2016, 2017a, 2017b).

To calculate admixture statistics, we used the R package admixr (Petr, Vernot, & Kelso, 2019) to run ADMIXTOOLS (Patterson et al., 2012). We converted our VCF file containing the Sitka deer (to use as an outgroup) into EIGENSTRAT format using a C++ script (found here: https://github.com/bodkan/vcf2eigenstrat). As the package does not work when including more than 600 scaffolds, we filtered the data set to include SNPs found only on the 600 largest scaffolds, which encompassed over 98% of the reference genome assembly (the scaffold L90 is 285; Taylor et al., 2019). We used the EIGENSTRAT files to run f3, f4 and f4‐ratio statistics. See Reich, Thangaraj, Patterson, Price, and Singh (2009) and Patterson et al. (2012) for full explanations of these tests, but briefly, the f3 statistic is a three‐population test that can calculate whether population ‘C’ is a mixture of two other populations, ‘A’ and ‘B’. A negative f3 statistic indicates that population ‘C’ is a mixture of ‘A’ and ‘B’. The f4 statistic is an ABBA BABBA test and acts similarly to D statistics. It is a four‐population test which requires a phylogenetic set‐up including two sister groups, a test group to see whether introgression has occurred into one of the two sister groups and an outgroup (which we always set as the Sitka deer). An f4 statistic which significantly differs from 0 indicates gene flow, whether it is positive or negative tells you into which of the sister populations. In the f4‐ratio test, alpha is calculated, which is the proportion of the genome in population ‘X’ that originates from population ‘B’ as opposed to population ‘A’ (the proportion of population ‘A’ is calculated as 1 – alpha).

For these tests, we grouped the four barrenground genomes from Bluenose and Qamanirijuaq as they show no differentiation and testing them separately made no difference to the results. The four boreal caribou genomes from Ontario and Manitoba were run separately as these do show differentiation and grouping them did affect the outcome. We focussed on using these tests to investigate 1) the amount of barrenground introgression into eastern migratory caribou in Ontario/Manitoba and Quebec/Labrador (f3, f4 and f4‐ratio tests) separately as they have nonoverlapping ranges, 2) introgression between eastern migratory caribou in Ontario/Manitoba and Quebec/Labrador (f4 test), 3) introgression between boreal caribou of NAL origin and the mountain caribou (f4 and f4‐ratio tests for significant populations) and 4) introgression between boreal caribou of NAL origin and the Northwest Territories boreal caribou of BEL origin (f4 and f4‐ratio tests), since one of our aims was to investigate the amount of introgression between the lineages and the potential role of adaptive introgression in leading to parallel evolution.

We first investigated introgression from barrenground into the Manitoba and Ontario boreal populations (f4 test), and due to its current geographical isolation and low levels of introgression from the barrenground lineage, we used Ignace as the representative NAL boreal population. Similarly, to investigate introgression from the NAL into the BEL, we used the Grant's caribou as the sister group as these showed the least amount of introgression from the NAL lineage (f4 test). In the tests to investigate BEL introgression into the boreal caribou of NAL origin, we used eastern migratory Quebec/Labrador caribou as the sister group which had the lowest introgression from the BEL (f3, f4 and f4‐ratio tests). For the full set‐up our tests, see Supporting Information.

To investigate patterns of introgression across the genome, we used the programme Dsuit (Malinsky, Matschiner, & Svardal, 2019). The Dinvestigate function can be used to calculate introgression in windows across the genome, and this was used to calculate the f_D_ and f_DM_ statistics (Malinsky et al., 2015) for sliding windows of 1,000 SNPs incremented by 250 SNPs across the genome. We used this programme to investigate introgression between NAL boreal caribou and the mountain caribou as well as between NAL boreal caribou and BEL boreal caribou to further investigate the process of parallel evolution. Again, we used Ignace as the representative NAL boreal population and as it is the most geographically isolated and has low levels of introgression from BEL. Similarly, we found Grant's caribou to show the lowest levels of introgression from the NAL and so we used them as the sister group into the BEL boreal and mountain caribou. The Sitka deer was still used as the outgroup in all tests.

To further investigate the potential role of adaptive introgression in the parallel evolution of the BEL boreal caribou (see Results), we investigated the gene composition of the most introgressed regions within the BEL boreal caribou identified as having originated from Ignace. We compared these to the most introgressed regions from Ignace into all mountain populations as adaptive introgression is unlikely to have played a role in the parallel evolution of these populations due to the uncovered patterns of introgression (see Results). To do this, we extracted the sequences for all regions across the genome with an f_DM_ score over 0.2 (as it is the most conservative statistic) using Bedtools 2.29 (Quinlan & Hall, 2010). To make sure the sister group used in the set‐up of the test did not bias the results, we only included regions that were flagged as highly introgressed from the NAL group when using both Grant's and Peary caribou as the sister group. We used the command line version of blast 2.6 (Altschul, Gish, Miller, Myers, & Lipman, 1990) to search for the genes present in these introgressed regions and genes with mRNA or predicted mRNA hits in at least two species and with an E score of 0.

## 3 RESULTS

### 3.1 Genome quality assessments

We sequenced 28 caribou genomes from across Canada and two caribou from Greenland to high coverage (35.57 – 43.03X; Table S1) representing all four subspecies and six Canadian designatable units (DUs; Figure 1 and Table 1) and used an additional reindeer genome from a domesticated animal in Inner Mongolia, sequenced by Li et al. (2017). Our caribou genomes showed high quality and recovery of BUSCO genes in the assembly, ranging from 92.7% to 93.1% of the more than 4,000 conserved genes surveyed (Table S1), and missing data per individual were low at 0.3%–1.0% with the exception of the previously published reindeer genome at 16.0% (Table S1).

### 3.2 Population and phylogenomic structure and demographic history

**FIGURE 2:**
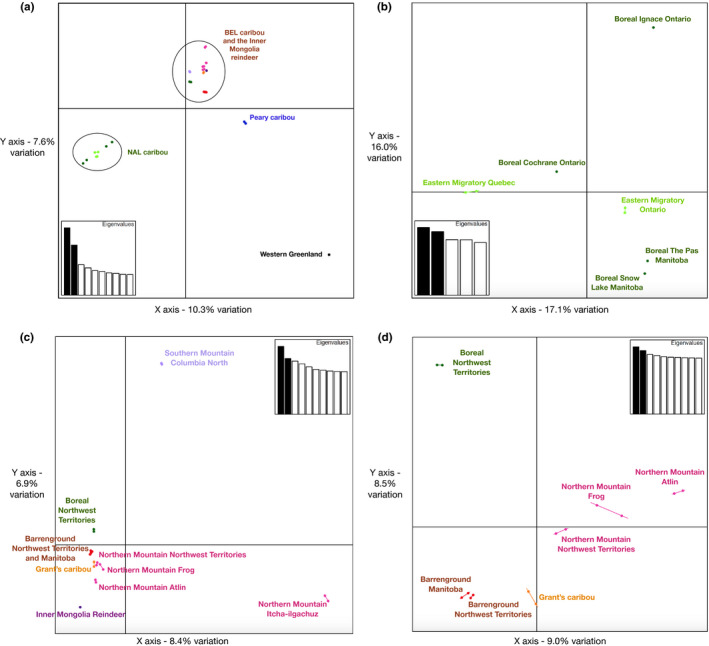
Principal component analyses of caribou genetic variation. All plots show PC1 (x‐axis) and PC2 (y‐axis) shown by the eigenvalues plot in the corners. The plots show PCA of all 30 caribou and the Inner Mongolian reindeer (a), fine‐scale analysis of the NAL caribou (b), fine‐scale analysis of the BEL caribou, aside from Peary and Western Greenland (c) and fine‐scale analysis of the 14 individuals clustered together from Figure 3c (d)[Colour figure can be viewed at wileyonlinelibrary.com]

Principal component analyses (PCAs) revealed four major clusters corresponding to the NAL and BEL as well as Peary and Greenland clusters (Figure 2a). The genome clustering did not conform to current subspecies or ecotype designations but did match previous mitochondrial results pertaining to the two refugial lineages (Figure 1; Table 1). Specifically, in the NAL cluster were some caribou populations of the woodland subspecies, that is boreal caribou from the eastern part of the range and eastern migratory caribou, but mountain caribou and boreal caribou from the Northwest territories grouped with Beringian lineages. The Beringian cluster contained barrenground, Grant's, northern mountain, southern mountain and Northwest Territory boreal caribou as well as the Inner Mongolian reindeer (Figure 2a). These lineages provide further evidence of the parallel evolution of similar ecotypes from distinct lineages and histories.

Finer resolution PCA of the NAL caribou showed all four boreal caribou were separated, particularly Ignace which may be due to genetic drift as it has a high inbreeding coefficient (Table S1; Figure 2b). Eastern migratory caribou from Ontario/Manitoba clustered closest to Manitoba boreal caribou although all were well separated (Figure 2b). Eastern migratory caribou from Quebec/Labrador were closest to Cochrane boreal and not eastern migratory caribou from Ontario/Manitoba and so indicate similar ecotypes may have evolved in parallel (Figure 2b). Fine‐scale analysis of the BEL caribou, aside from Peary and Western Greenland, showed the Inner Mongolian reindeer and northern mountain caribou from Itcha‐Ilgachuz separating, which again may be due to drift and inbreeding (Table S1; Figure 2c). Southern mountain caribou from Columbia North are also relatively well separated. The rest all formed a relatively tight cluster, with the Northwest Territories boreal caribou and the Grant's caribou slightly separated (Figure 2c). We ran the 14 genomes that sat closely together in another PCA and found the four barrenground caribou clustered together and the others to separate, especially the Northwest Territories boreal (Figure 2d).

**FIGURE 3:**
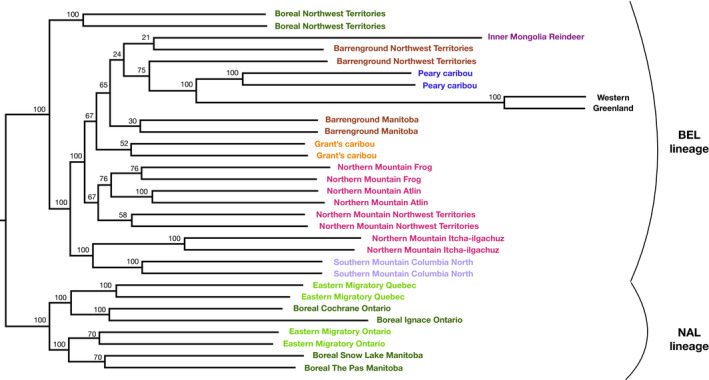
Maximum‐likelihood phylogenomic reconstruction from SNP data in RAxML of 30 caribou and the Inner Mongolian reindeer. We show the unrooted phylogeny for clarity, with the root fixed where indicated in analyses using the Sitka deer as an outgroup (See Figure 5 and Figure S8). Nodes show bootstrap support values[Colour figure can be viewed at wileyonlinelibrary.com]

**FIGURE 4:**
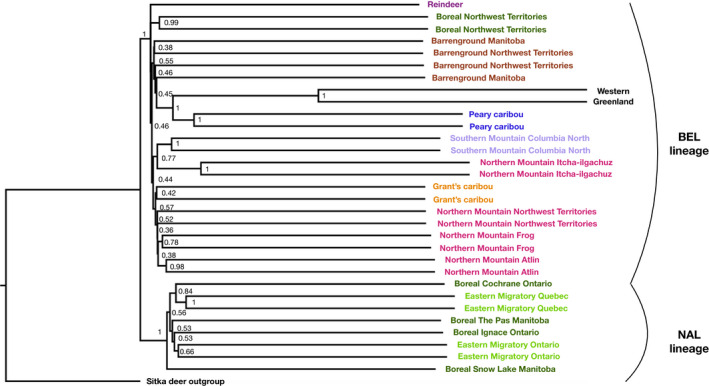
Consensus maximum‐likelihood phylogenomic reconstruction from ~4,000 conserved mammalian gene sequences from BUSCO of 30 caribou and the Inner Mongolia reindeer rooted using a Sitka deer outgroup. Nodes show bootstrap support values[Colour figure can be viewed at wileyonlinelibrary.com]

Phylogenomic reconstruction using SNPs in RAxML (Figure 3) and conserved gene sequences from BUSCO (Figure 4) showed similar patterns. Both separated the NAL lineage from all others and within the NAL clade eastern migratory caribou from Ontario/Manitoba and those from Quebec/Labrador were not reconstructed as sister groups, again indicating parallel evolution of the eastern migratory ecotype. In the SNP phylogeny, which has been rooted based on the BUSCO phylogeny (Figure 4) and the Treemix analysis with the Sitka deer (Figure S8), within the NAL clade eastern migratory caribou from Quebec are reconstructed as sister to boreal caribou from Ontario, whereas eastern migratory caribou from Ontario were placed as sister to boreal caribou from Manitoba which matches the geography of the sampling locations (Figure 1; Figure 3). Within the BEL clade, the boreal caribou from the Northwest Territories are reconstructed as basal to all others. The rest were split into two clades, one of these with Northern mountain caribou from Itcha‐Ilgachuz and the southern mountain Columbia North caribou. The other clade was further split into two, with the northern mountain caribou from the Northwest Territories, Atlin and Frog in one, and the other with the barrenground caribou from the Northwest Territories and Manitoba, Grant's caribou, the Inner Mongolia reindeer and Peary and Western Greenland caribou forming a sister clade within the group (Figure 3).

The rooted BUSCO phylogeny shows similar patterns to the SNP reconstruction although with shorter branch lengths between groups and lower support of nodes which is unsurprising given that is was reconstructed from conserved mammalian genes and so does not give as much resolution for the fine‐scale structure. Importantly, the analysis also separated the NAL and BEL clades with high support (Figure 4) as with the SNP tree (Figure 3). As with the SNP phylogeny, the Northwest Territories boreal and all mountain caribou sat within the BEL clade as further evidence for parallel evolution of the woodland ecotype.

**FIGURE 5:**
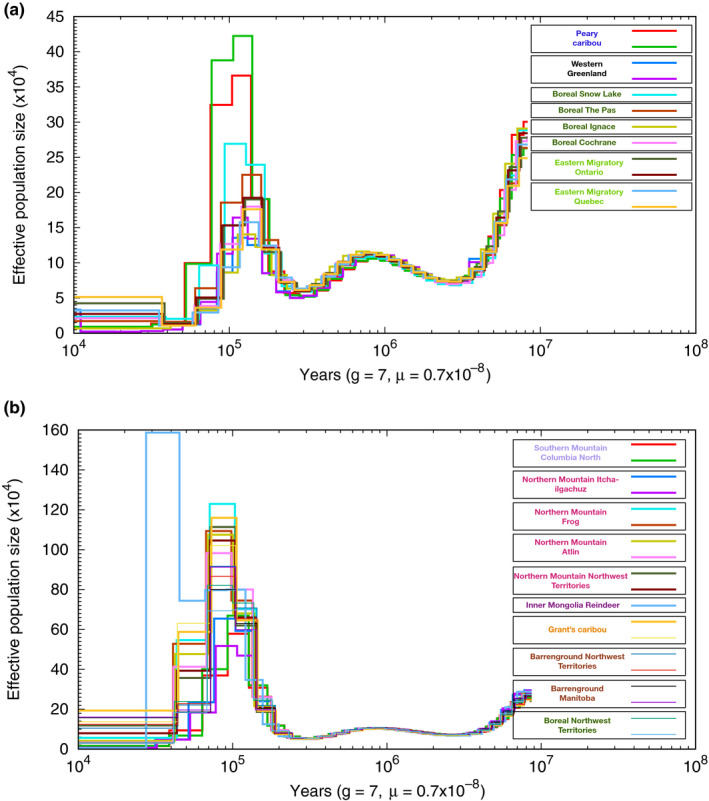
Reconstruction of effective population size of caribou. Results have been split into two plots given the differences in peak effective population sizes, with (a) showing the NAL lineage and Peary and Western Greenland caribou and (b) showing all other BEL caribou. The effective population sizes remain the same until 100–200 kya where demographic histories start to differ, with peak population sizes 120 kya. NAL caribou have smaller peak effective population sizes than BEL caribou, with Peary and Greenland caribou intermediate[Colour figure can be viewed at wileyonlinelibrary.com]

A reconstruction of caribou demography over time using PSMC indicated a major population size expansion starting approximately 100–200 kya with peak population sizes around the glacial interstitial stage of a largely ice‐free North America 120 kya (Figure 5a,b). This timing corresponds to a divergence of lineages largely concordant with the expansion and intraspecific diversification proposed by Banfield (1961). The NAL and the Greenland caribou have much lower population sizes during the expansion 100–200 kya than BEL caribou, including the boreal caribou from the Northwest Territories, with Peary caribou being intermediate to these groups (Figure 5a,b, Figure S6 for all plotted together) consistent with an earlier divergence (Klütsch, Manseau, Anderson, Sinkins, & Wilson, 2017). Population sizes for all caribou lineages declined during the Wisconsin glaciation which lasted between 75 and 11 kya (Figure 5a,b), with the exception of the reindeer which has a unique demographic trajectory likely as a result of domestication.

Contemporary inbreeding estimates varied greatly between different individuals. For the North American caribou, they ranged from −0.009 to 0.311. They were highest for Greenland at 0.654, and the Inner Mongolia reindeer also had an elevated coefficient at 0.177, again reflecting the origin of the latter as originating from a domesticated population (Li et al., 2017; Table S1).

### 3.3 Patterns of introgression

**FIGURE 6:**
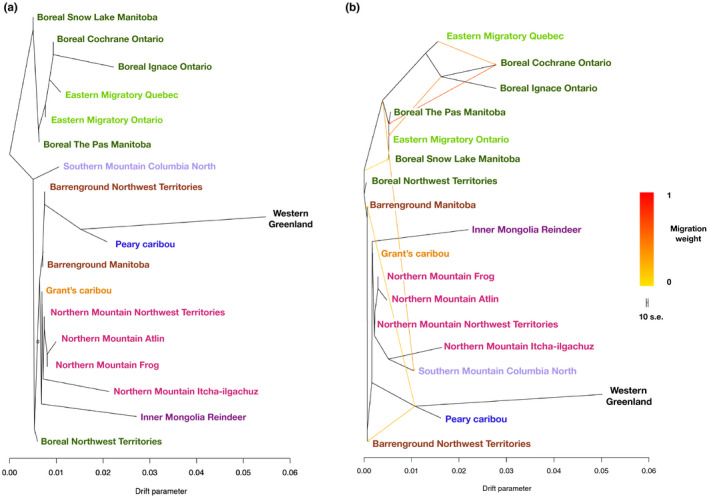
Unrooted maximum‐likelihood phylogeny reconstructed in Treemix with no migration events added (a) and an unrooted maximum‐likelihood phylogeny reconstructed in Treemix with 7 migration events added (b) as indicated from the OptM results (Figure S7)[Colour figure can be viewed at wileyonlinelibrary.com]

To assess the contribution of admixture and introgression among lineages in positioning caribou lineages, we applied Treemix and f3, f4 and f4‐ratio statistics. The Treemix phylogeny with no migration events gave a similar topology to the RAxML tree (Figure 6a). When visualizing seven migration events, which shows the least standard error and has the highest delta m score (Figure S7), we see migration from the ancestor of Peary and Western Greenland into both Northwest Territories and Manitoba barrenground, and a migration event from the ancestor of the NAL lineage into southern mountain caribou (Figure 6b). The other migration events all occur within the NAL group, including into Snow Lake from an ancestral group, from the ancestor of Cochrane and Ignace into Eastern migratory Ontario, from Cochrane into an ancestor of Snow Lake and The Pas and Eastern migratory Ontario and from Eastern migratory Quebec into Cochrane. The tree shows large drift parameters for those individuals with high inbreeding coefficients (Table S1; Figure 6b).

The f3 results indicated that the genomes of eastern migratory caribou in Ontario/Manitoba resulted from admixture between NAL boreal caribou from Igance (our reference NAL genome, see Methods) and barrenground caribou, as well as from between NAL boreal caribou from Igance and the other genomes from the BEL (Table 2). This indicates that eastern migratory caribou from Ontario/Manitoba have had introgression from the BEL lineage, in agreement with previous findings (Klütsch et al., 2016). The f4‐ratio statistic showed the Ontario/Manitoba eastern migratory caribou genomes to be of 7% barrenground origin (see Supporting Information for all statistics). In contrast, there were no negative f3 scores for eastern migratory caribou in Quebec/Labrador, including from barrenground, with no proportion of their genome of barrenground origin (Table 3). These results indicate that the eastern migratory caribou from the two disjunct ranges have different demographic histories. Given the f3 results, we used the f4 statistic to test for introgression between the disjunct eastern migratory caribou populations and found evidence for introgression from Quebec into Ontario/Manitoba eastern migratory but not the other way around (Supporting Information).

**TABLE 2:** f3 statistic results testing whether eastern migratory caribou from Ontario/Manitoba show signatures of admixture between boreal Ignace caribou and populations from the BEL lineage.

BEL population	f_3_ statistic	Standard error	Z score
Barrenground	−0.016	0.006	−2.628
Southern mountain Columbia North	−0.016	0.006	−2.6
Northern mountain Itcha‐Ilgachuz	−0.015	0.007	−2.219
Northern mountain Frog	−0.016	0.007	−2.484
Northern mountain Atlin	−0.016	0.006	−2.657
Northern mountain Redstone	−0.018	0.007	−2.548
Northwest Territories boreal	−0.017	0.007	−2.349
Grant's caribou Porcupine	−0.015	0.006	−2.276
Peary caribou	−0.017	0.007	−2.567
Western Greenland	−0.022	0.007	−3.315
Inner Mongolian reindeer	−0.019	0.006	−3.071

**TABLE 3:** f3 statistic results testing whether eastern migratory caribou from Quebec/Labrador show signatures of admixture between boreal Ignace caribou and populations from the BEL lineage.

BEL population	f_3_ statistic	Standard error	Z score
Barrenground	0.013	0.010	1.335
Southern mountain Columbia North	0.011	0.009	1.217
Northern mountain Itcha‐Ilgachuz	0.012	0.008	1.445
Northern mountain Frog	0.014	0.008	1.675
Northern mountain Atlin	0.012	0.009	1.334
Northern mountain Redstone	0.011	0.008	1.366
Northwest Territories boreal	0.012	0.009	1.427
Grant's caribou Porcupine	0.013	0.009	1.475
Peary caribou	0.013	0.009	1.477
Western Greenland	0.004	0.009	0.445
Inner Mongolian reindeer	0.004	0.009	0.45

The f4 results did not show introgression from Northwest Territories boreal, southern mountain Columbia North or any northern mountain population into NAL boreal caribou (full results for these tests in Supporting Information). The f4 results showed signatures of introgression from the NAL boreal caribou into southern mountain Columbia North (Table 4), with the f4‐ratio statistic indicating that 13.3% of their genomes shows NAL boreal origin. However, we find no strong evidence of introgression from NAL boreal into any of the northern mountain caribou (Table 4). The f4 results do indicate strong signatures of introgression from the NAL boreal caribou into Northwest Territories boreal caribou (Table 4), with the f4‐ratio test suggesting that 16.2% of their genomes originates from the NAL boreal caribou, indicating the possibility of parallel evolution of the same ecotype by adaptive introgression (Supporting Information).

**TABLE 4:** f4 statistics testing for introgression from boreal Ignace caribou (population ‘C’) into mountain caribou, Northwest Territories boreal caribou and barrenground caribou populations (population ‘A’). Grant's caribou was used as the sister population (population ‘B’) for all tests and Sitka deer as the outgroup (population ‘D’).

BEL population	f_4_ statistic	Standard error	*Z* score	BABA	ABBA
Barrenground	0.0006	0.0002	3.772	21,215	20,246
Southern mountain Columbia North	0.0007	0.0003	2.535	21,452	20,291
Northern mountain Itcha‐Ilgachuz	0.0004	0.0002	1.574	21,092	20,489
Northern mountain Frog	0.0003	0.0003	0.935	21,010	20,533
Northern mountain Atlin	0.0000	0.0002	0.159	20,424	20,373
Northern mountain Redstone	0.0000	0.0002	0.127	20,301	20,265
Northwest Territories boreal	0.0010	0.0003	3.851	21,449	19,822

We also used average genomewide f_D_ and f_DM_ statistics to estimate the proportion of the genome resulting from introgression, comparable to the f4‐ratio scores, and we found the same trends although generally lower with the f_DM_ statistic, likely because it is a conservative estimate (full results in Supporting Information).

To investigate the possibility of adaptive introgression in the parallel evolution of the Northwest Territories boreal caribou, we looked at patterns of introgression across the genome and investigated the gene compliment of the most highly introgressed regions. The neutral expectations of these patterns are unknown, but we compared the results with all mountain caribou populations to see whether there is a difference in the overall genomewide pattern and the number of coding regions being introgressed. The mountain caribou are unlikely to have undergone adaptive introgression in the process of parallel evolution given that they have varying levels of introgression, with those closest to the Northwest Territories boreal caribou having negligible levels. When looking at the f_DM_ statistics in sliding windows across the genome, and for all comparisons, the regions of introgression appeared spread out throughout the genome encompassing both neutral and functional sites (Figures S9–S14). Within the most highly introgressed regions from the NAL boreal caribou, with an f_DM_ score of at least 0.2, we found 49 highly introgressed regions (f_DM_ 0.2 or above) originating from the NAL into Northwest Territories boreal caribou. Within these regions, there were a total of 118 genes, with an average of 2.46 genes per introgressed region (Supporting Information for regions and gene lists). In the southern mountain Columbia North population, which is closest geographically to the boreal populations out of our sample locations and has very similar overall levels of introgression as Northwest Territories boreal caribou, we find 64 comparable regions, containing 244 genes and an average of 3.81 genes per region. The northern mountain populations all have fewer of these large, highly introgressed regions as expected from their overall very low levels of introgression from NAL; however, the few genomic regions which have introgressed do also contain numerous gene sequences (Itcha‐Ilgachuz 14 regions with 39 genes and an average of 2.79 genes per region; Frog has six regions with 18 genes and an average of three genes per region; Atlin has eight regions with 26 genes and an average 3.25 genes per region; Redstone in the Northwest Territories has nine regions with 44 genes and an average 4.89 genes per region; see Supporting Information for regions and gene lists).

## 4 DISCUSSION

Genome sequences of 30 caribou from across North America and Greenland provided a comprehensive data set in a nonmodel terrestrial mammal species at risk. We reconstructed phylogenomic and demographic history and measured levels of introgression between ecotypes and investigated the potential role this introgression has played in parallel evolution. Our results are concordant with previous mtDNA studies (Cronin et al., 2005; Flagstad & Røed, 2003; Klütsch et al., 2012, 2016; Weckworth et al., 2012) which found two major mitochondrial DNA phylogenetic lineages, NAL and BEL, which likely correspond to divergence within refugia during glacial cycles (Flagstad & Røed, 2003; Weckworth et al., 2012), as per our first aim. This reflects why the Inner Mongolia reindeer is uncovered as within the BEL lineage as it likely resided in the same Beringian refugia during glacial maxima. We found Peary caribou to be genetically distinct from the others in the BEL lineage (Figure 2a), supporting previous evidence of an additional High Arctic refugium (Klütsch et al., 2017). Greenland were recovered as a sister group to the Peary caribou within the BEL linage, but were also genetically distinct (Figure 2a).

Inbreeding coefficients varied greatly between sampling locations. Peary and Greenland caribou have elevated inbreeding coefficients (Table S1) likely reflecting recent bottlenecks in those populations (Jepsen, Siegismund, & Fredholm, 2002; Taylor, Jenkins, & Arcese, 2012), and genetic drift may have driven their separation in the PCA (Figure 2a). Inbreeding coefficients were also elevated for boreal caribou from Ignace and northern mountain caribou from Itcha‐Ilgachuz which may be due to geographical isolation of those populations (Figure 1). Demographic reconstruction over time showed differential population trajectories of the lineages starting approximately 100–120 kya, indicating divergence to have started well before the last glacial maximum approximately 19–27 kya (Figure 5a,b; Clark et al., 2009).

### 4.1 Parallel evolution and introgression in caribou ecotypes

As per our second aim, our results confirm previous evidence that northern mountain and boreal caribou from the Northwest Territories are within the BEL genomic lineage, even though they are both currently within the woodland subspecies alongside the boreal caribou from the east and central part of their range which are in the NAL genomic lineage. This confirms that the woodland ecotype appears to have arisen in parallel for both (Polfus et al., 2017). Our central mountain caribou are also within the BEL genomic lineage, and this population has been found to be a mixture of the two mtDNA lineages (McDevitt et al., 2009). Within the NAL lineage, we found evidence for another, as yet undocumented, case of parallel evolution within the eastern migratory ecotype. The eastern migratory caribou from Ontario/Manitoba and those from Quebec/Labrador are not sister groups (Figures 2b and 3) and have different demographic and introgressive histories.

Recent studies are highlighting that introgression between lineages is far more common than previously realized (Coates, Byrne, & Moritz, 2018; Hamilton & Miller, 2015), and the same appears to be true for caribou with introgression likely playing a role in the evolution of the ecotypes. For our third and fourth aims, we investigated patterns of genomewide introgression among the caribou lineages and we found some populations to have high levels of introgression; for example, the barrenground caribou have substantial introgression from the NAL. We support previous results indicating that eastern migratory caribou from Ontario/Manitoba appear to be admixed between NAL boreal and barrenground caribou (Table 2); however, we do not find the same pattern for the eastern migratory caribou from Quebec/Labrador (Table 3). It seems unlikely that adaptive introgression drove the parallel evolution of the eastern migratory phenotype given the lack of gene flow into the Quebec/Labrador population, although this is difficult to test with their shared phylogenomic history (they are both within the NAL clade) and probable high levels of incomplete lineage sorting (ILS).

Introgression is also seen from the NAL into some of the mountain caribou, with negligible levels of introgression detected into more northerly northern mountain caribou. It thus seems unlikely that introgression drove the parallel evolution of the woodland phenotype of the mountain caribou. We find high levels of introgression and many introgressed genes from the NAL lineage into the Northwest Territories boreal caribou. However, when we compare the gene compliment of the most highly introgressed regions in the Northwest Territories boreal caribou to those found in the mountain caribou, we again find introgressed regions spread across the genome including many genes, even though there are fewer regions overall. There are a few explanations for this pattern, including ILS. ILS would be difficult to exclude, especially given that they are closely related intraspecific ecotypes (Lamichhaney et al., 2017). Whether these regions are a result of ILS or introgression the high gene compliment suggests that they could have persisted in the genome due to selection, even if they have not been involved in the parallel evolution of phenotype, due to filtration for maintenance of adaptive genome segments. Additionally, when studying cases of adaptive introgression in interspecies comparisons, areas of introgression are often restricted to single genomic regions (Schweizer et al., 2019); however, in intraspecific taxa, we may see larger introgressed regions persisting across the genome because the fitness costs may be lessened.

Alternatively, given the genomewide patterns of the introgressed regions (Figures S9–S14), a likely explanation for the variation in admixture could be genetic drift. As we see introgression ‘peaks’ throughout the genome, encompassing neutral and functional regions in the Northwest Territories boreal caribou and all mountain caribou, neutral rather than adaptive processes may be the primary driver. This would make it seem likely that adaptive introgression did not drive the parallel evolution but standing variation may be most likely. Fully teasing these patterns apart in this case may be complicated because multiple processes are likely acting in concert, including ILS, genetic drift and standing variation being selected upon, coupled with differing levels of introgression as the lineages have come into secondary contact. Additionally, given the PSMC results, it is possible that there have been multiple bouts of introgression during glacial cycles over the last ~120,000 years if the lineages repeatedly came into secondary contact, something which needs to be investigated. Demonstrating adaptive introgression is complicated and requires the demonstration of the adaptive function of introgressed regions, meaning most cases have thus far been for well understood traits or those controlled by a single locus (Taylor & Larson, 2019). In contrast, investigating parallel evolution of ecotypes, which will inevitably involve many functional regions, in a nonmodel species with divergent intraspecific linages and complex demographic histories is a difficult task.

### 4.2 Conservation unit designations in the light of complex demographic histories

Given current rates of extirpation and extinction, it is imperative to have strong, scientifically supported management frameworks, particularly given tight resources for conservation (Jackiw, Mandil, & Hager, 2015). Recent work shows that admixture between lineages is common (Coates et al., 2018; vonHoldt et al., 2018) and that new sequencing technologies are allowing us to uncover the complex demographic histories of threatened taxa (Supple & Shapiro, 2018; vonHoldt et al., 2018). Both for caribou and more broadly, now is the time to decide what this means for management and conservation unit designations.

Recent discussion has highlighted that even high levels of gene flow are not always negative, particularly in inbred populations or those needing to adapt to rapid change where admixture could be an important source of variation (Supple & Shapiro, 2018; vonHoldt et al., 2018). For example, we find the barrenground caribou to be admixed and also to have the lowest individual inbreeding coefficients, and similarly, eastern migratory caribou from Ontario/Manitoba have lower inbreeding coefficients than the nonadmixed individuals from Quebec/Labrador (Table S1). Some argue that gene flow could even be facilitated to aid populations under threat from climate change (or an increase in the fitness of a population due to the introduction of new alleles, that is genetic rescue; Hamilton & Miller, 2015; Whiteley, Fitzpatrick, Funk, & Tallmon, 2015), which would be easiest between intraspecific populations (Hedrick & Fredrickson, 2010). Good conservation unit designations with an understanding of natural patterns of admixture are key to assess the potential to use such a strategy (Coates et al., 2018). Outbreeding depression is a potential issue when thinking about a genetic rescue strategy and conservation unit designations containing genetically distinct lineages which were grouped due to parallel evolution, as we see here in the caribou, could present a problem. When considering admixed populations, most discussions have focussed on policy for interspecies hybridization (but see Coates et al., 2018; Supple & Shapiro, 2018), but a clear framework for conservation unit designation of admixed intraspecific lineages is needed. Additionally, human‐induced hybridization and introgression are emerging issues for conservation and are increasing (Allendorf, Leary, Spruell, & Wenburg, 2001); however, here we are focussing on introgression driven by historical processes.

Conservation unit designations depend on the goal of conservation, and whether the focus is on the preservation of phenotypes (or ‘pure’ genomes), or evolutionary and ecological processes to maintain resilience of an ecosystem (Fitzpatrick et al., 2015; vonHoldt et al., 2018; Waples & Lindley, 2018). The latter is likely more useful when attempting to designate units for nondiscrete entities, such as we see in caribou. With this in mind, some authors have suggested a flexible approach with each case considered on a context‐specific basis (Jackiw et al., 2015), whereas others promote the need for a structured and uniform framework to decide on management decisions (Coates et al., 2018). For caribou, and other taxa, it seems appropriate for a structured approach in the naming of subspecies. Coates et al. (2018) suggest that subspecies show local adaptation with or without gene flow. Coupling this idea with our phylogenomic and population genomic results and results from previous studies, Canadian caribou appear to fit into three subspecies; those in the NAL, those in the BEL, and Peary caribou which sit phylogenetically in the BEL but show strong population genomic differences and clear local adaptation of phenotype (Banfield, 1961; COSEWIC, 2011).

The most relevant application of our findings is in the delineation of conservation units in a species with complex and admixed evolutionary histories. We recommend that previously defined designatable units based on subspecies and subspecific ecotypes be reconsidered: specifically, because the boreal caribou from the Northwest Territories sit within a different lineage to the other caribou within the boreal DU and appear to have evolved in parallel, they could be split into separate DUs. Further fine‐scale work will be needed to refine the boundary of the BEL boreal versus the NAL boreal DU. Similarly, given the apparent parallel evolution of the eastern migratory ecotype and the different levels of admixture of Ontario/Manitoba versus Quebec/Labrador populations with the BEL lineage, the eastern migratory ecotype should perhaps be divided into separate DUs. Consideration of whether this will help maximize the resilience of the ecosystem is needed, but this would match the evolutionary processes which have led to the evolution of the groups. Confusingly, Grant's caribou and barrenground caribou are currently separate subspecies but one DU. Barrenground caribou are admixed which contrasts with the Grant's caribou we sampled and so perhaps they warrant listing as separate DUs. Further sampling is needed to resolve the mountain caribou, especially the central mountain population which has been shown to have mitochondrial DNA from both the BEL and NAL lineages (McDevitt et al., 2009). Additionally, genomic data from the southern mountain, and all other DUs not included in this study, are needed to further resolve the complex evolutionary histories and patterns of introgression more broadly. These divisions have significant implications for the status listing of each DU as threat status is assessed based on criteria such as abundance, and priority for management is given to DUs at greatest risk of extinction (COSEWIC, 2015). Given recent rapid declines in both range and population sizes, efficient conservation strategies are needed for caribou.

Our guidelines add to the current discussion about management of admixed populations and those with complex demographic histories (Coates et al., 2018; Fitzpatrick et al., 2015; Hamilton & Miller, 2015; Jackiw et al., 2015; Supple & Shapiro, 2018; vonHoldt et al., 2018). Namely, that subspecies designations are useful and could follow a structured framework (Coates et al., 2018), but that conservation units below the subspecies level likely require a case‐by‐case consideration especially given different regulations in different countries (Coates et al., 2018; vonHoldt et al., 2018). Genomic data allow detailed investigations of demographic histories and genomewide patterns of introgression, and these results should be considered in conservation unit designations to make meaningful management decisions. Many taxa are facing an increasing threat from climate change and habitat destruction (Hoffman, Sgrò, & Kristensen, 2017; Ikeda et al., 2017) and genomic data and appropriate conservation unit designations will help with prioritization given limited resources. This means considering whether taxa need dividing into different units to ensure populations with different evolutionary histories and trajectories are maintained, or whether they should be kept as one conservation unit as over splitting could spread resources too thinly. For example, this decision will need to be made for eastern migratory caribou. A key next step to achieve these goals, including for caribou, is to use the wealth of data available from genomewide markers to investigate adaptive genomic variation to incorporate with demographic history information (Funk, Forester, Converse, Darst, & Moreys, 2019; Funk, McKay, Hohenloe, & Allendorf, 2012).

## AUTHOR CONTRIBUTIONS

R.S.T., R.L.H., M.M. and P.J.W. conceived and designed the study. R.S.T., R.L.H. and S.K. performed bioinformatics analyses. R.S.T. wrote the manuscript, and R.L.H., M.M., G.B.G. and P.J.W provided feedback and edited the manuscript.

## Supporting information

Supplementary Material

Supplementary Material

Supplementary Material

Supplementary Material

Supplementary Material

Supplementary Material

## Data Availability

All raw sequencing data are available at the National Centre for Biotechnology (NCBI) under the BioProject Accession no. PRJNA634908 (Taylor et al., 2020).
