# Supplementary material for: The role of introgression and ecotypic parallelism in delineating intraspecific conservation units
Source: Mol Ecol. 2020 Jul 11;29(15):2793–809. doi: 10.1111/mec.15522 (PMC7496186; doi:10.1111/mec.15522)
Supplement: Supplementary file 1 — Supplementary Material [file MEC-29-2793-s001.pdf]

MOLECULAR ECOLOGY

Supplemental Information for:

The role of introgression and ecotypic parallelism in delineating intra-specific conservation units

Rebecca S. Taylor, Micheline Manseau, Rebekah L. Horn, Sonesinh Keobouasone, G. Brian Golding, Paul J. Wilson

Table of Contents:

**Table S1**	Page 2
**Figure S1**	Page 6
**Figure S2**	Page 7
**Figure S3**	Page 8
**Figure S4**	Page 9
**Figure S5**	Page 10
**Figure S6**	Page 11
**Figure S7**	Page 12
**Figure S8**	Page 13
**Figure S9**	Page 14
**Figure S10**	Page 15
**Figure S11**	Page 16
**Figure S12**	Page 17
**Figure S13**	Page 18
**Figure S14**	Page 19
**Supporting Information f3 tests**	Spreadsheet f3_SitkaOutgroup
**Supporting Information f4 tests**	Spreadsheet f4_SitkaOutgroup
**Supporting Information f4-ratio**	Spreadsheet 4_ratio_SitkaOut
**Supporting Information genome-wide f_D and f_{DM} statistics**	Spreadsheet Dsuit_Genomewide_Stats
**Supporting Information Introgressed regions and gene lists**	Spreadsheet Dinvestigate

MOLECULAR ECOLOGY

Table S1. Information for each caribou including sampling location and Individual ID, collection year and tissue type, mean depth of coverage from the BAM file, the number and percentage of BUSCO genes reconstructed in total and as a single copy, mean depth in the VCF file, missing data, and individual inbreeding co-efficients. Samples were collected on road kills or from harvested animals by biologists or veterinarians with the British Columbia, Manitoba, and Ontario provincial governments, the Canadian federal government, the Greenland government, the Sahtú Renewable Resources Board, The Royal Ontario Museum, the University of Manitoba, and an independent consultant

Individual	Collection Information	Tissue Type	Mean Depth (BAM file)	Complete BUSCO genes recovered	Complete and single copy BUSCO genes recovered	Mean depth after filtering (VCF file)	Mean missing data after filtering (VCF file)	Inbreeding co-efficient, F
Northwest Territories, Redstone herd, Northern mountain caribou 15460	2013	Muscle	47.034	3,814 (92.9%)	3,789 (92.3%)	38.099	0.004	0.002
Northwest Territories Sahtú region Boreal caribou 17825	2013	Muscle	45.234	3,812 (92.9%)	3,788 (92.3%)	36.915	0.004	0.036
Northwest Territories, Redstone herd, Northern mountain caribou 17896	2014	Muscle	43.891	3,818 (93.0%)	3,794 (92.4%)	35.563	0.005	0.007
Ontario Pen Island herd, Eastern migratory caribou 20917	Unknown	Hide	39.606	3,816 (93.0%)	3,789 (92.3%)	33.050	0.008	0.077
Manitoba Qamanirijuaq herd, Barrenground caribou 21332	2008	Hide	42.258	3,813 (92.9%)	3,790 (92.3%)	34.933	0.006	-0.005

Manitoba Qamanirijuaq herd, Barrenground caribou 21350	2008	Hide	41.879	3,821 (93.1%)	3,798 (92.5%)	34.467	0.007	-0.009
Northwest Territories Bluenose herd Barrenground caribou 27177	2013	Muscle	46.617	3,819 (93.1%)	3,792 (92.4%)	37.888	0.004	-0.002
Northwest Territories Bluenose herd Barrenground caribou 27186	2013	Muscle	43.403	3,817 (93.0%)	3,795 (92.5%)	35.757	0.005	-0.001
Quebec George River herd, Eastern migratory caribou 27689	2008	Muscle	42.165	3,808 (92.8%)	3,781 (92.1%)	34.705	0.007	0.131
Quebec George River herd, Eastern migratory caribou 27694	2008	Muscle	42.900	3,820 (93.1%)	3,793 (92.4%)	35.031	0.007	0.105
Yukon Porcupine herd, Grant's caribou 27737	2001	Muscle	44.510	3,808 (92.8%)	3,783 (92.2%)	36.496	0.004	-0.001
Yukon Porcupine herd, Grant's caribou 27738	2001	Muscle	45.113	3,812 (92.9%)	3,788 (92.3%)	36.904	0.003	0.007
British Columbia Frog herd, Northern mountain caribou 28327	2002	Hide	39.019	3,811 (92.9%)	3,784 (92.2%)	32.716	0.005	0.014
British Columbia Frog herd, Northern mountain caribou 28337	2003	Hide	42.117	3,807 (92.7%)	3,781 (92.1%)	35.055	0.004	0.047
British Columbia Itcha-Ilgachuz herd,	2006	Hide	36.077	3,815 (92.9%)	3,790 (92.3%)	30.362	0.007	0.208

Northern mountain caribou 28395								
British Columbia Itcha-Ilgachuz herd, Northern mountain caribou 28402	2006	Hide	39.496	3,817 (93.0%)	3,793 (92.4%)	33.056	0.006	0.195
British Columbia Atlin herd, Northern mountain caribou 28575	2006	Hide	40.334	3,824 (93.1%)	3,798 (92.5%)	33.436	0.006	0.028
British Columbia Atlin herd, Northern mountain caribou 28580	2006	Hide	40.009	3,818 (93.0%)	3,796 (92.5%)	33.295	0.005	0.042
British Columbia Columbia North herd, Southern mountain, caribou 28646	2014	Hide	41.056	3,818 (93.0%)	3,789 (92.3%)	33.524	0.005	0.086
British Columbia Columbia North herd, Southern mountain, caribou 28649	2014	Hide	41.109	3,815 (92.9%)	3,789 (92.3%)	33.655	0.006	0.085
Nunavut Bathurst Island Peary caribou 34549	1993	Muscle	45.421	3,818 (93.0%)	3,796 (92.5%)	36.882	0.005	0.209
Nunavut Bathurst Island Peary caribou 34550	1993	Muscle	45.282	3,815 (92.9%)	3,793 (92.4%)	36.494	0.006	0.202
Ontario Pen Islands herd, Eastern migratory caribou 34590	1992	Muscle	40.863	3,813 (92.9%)	3,787 (92.3%)	33.943	0.006	0.098

MOLECULAR ECOLOGY

Northwest Territories Sahtú region Boreal caribou 35082	2015	Muscle	44.095	3,818 (93.0%)	3,798 (92.5%)	36.391	0.005	0.037
Manitoba Naosap herd (The Pas), Boreal caribou 35324	Road kill 2008	Muscle	39.264	3,821 (93.1%)	3,797 (92.5%)	32.417	0.007	0.112
Manitoba Naosap herd (Snow Lake), Boreal caribou 35326	Road kill 2009	Muscle	42.456	3,822 (93.1%)	3,797 (92.5%)	34.944	0.003	0.080
Ontario Ignace, Boreal caribou 39590	Unknown	Muscle	37.678	3,821 (93.1%)	3,794 (92.4%)	31.159	0.010	0.311
Ontario Cochrane Boreal caribou 39654	2009	Muscle	43.314	3,809 (92.8%)	3,781 (92.1%)	35.819	0.003	0.130
Western Greenland Kangerlussuaq 41660	2009	Kidney	40.299	3,817 (93.0%)	3,790 (92.3%)	33.288	0.007	0.654
Western Greenland Kangerlussuaq 41667	2009	Kidney	35.574	3,818 (93.0%)	3,793 (92.4%)	29.932	0.008	0.654
Inner Mongolia domesticated Reindeer	See Li et al. 2019	See Li et al. 2019	180.889	3,819 (93.0%)	3,793 (92.4%)	151.909	0.162	0.177
Sitka deer			22.167	3,804 (92.7%)	3,781 (92.1%)			n/a

MOLECULAR ECOLOGY

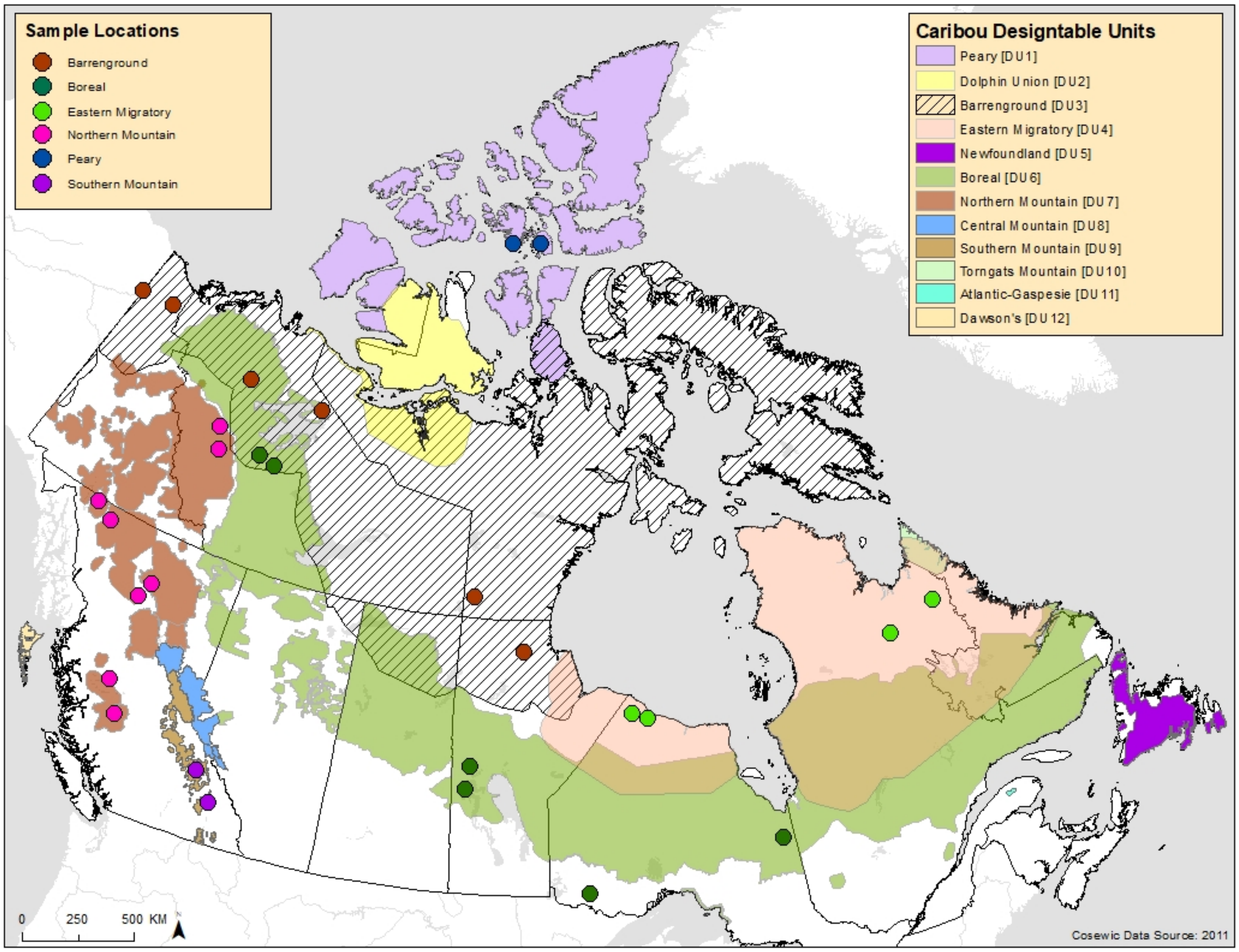

Figure S1 Distribution of the 12 Designatable Units (DUs) under COSEWIC (2011). Samples used in this study are indicated in circles.

MOLECULAR ECOLOGY

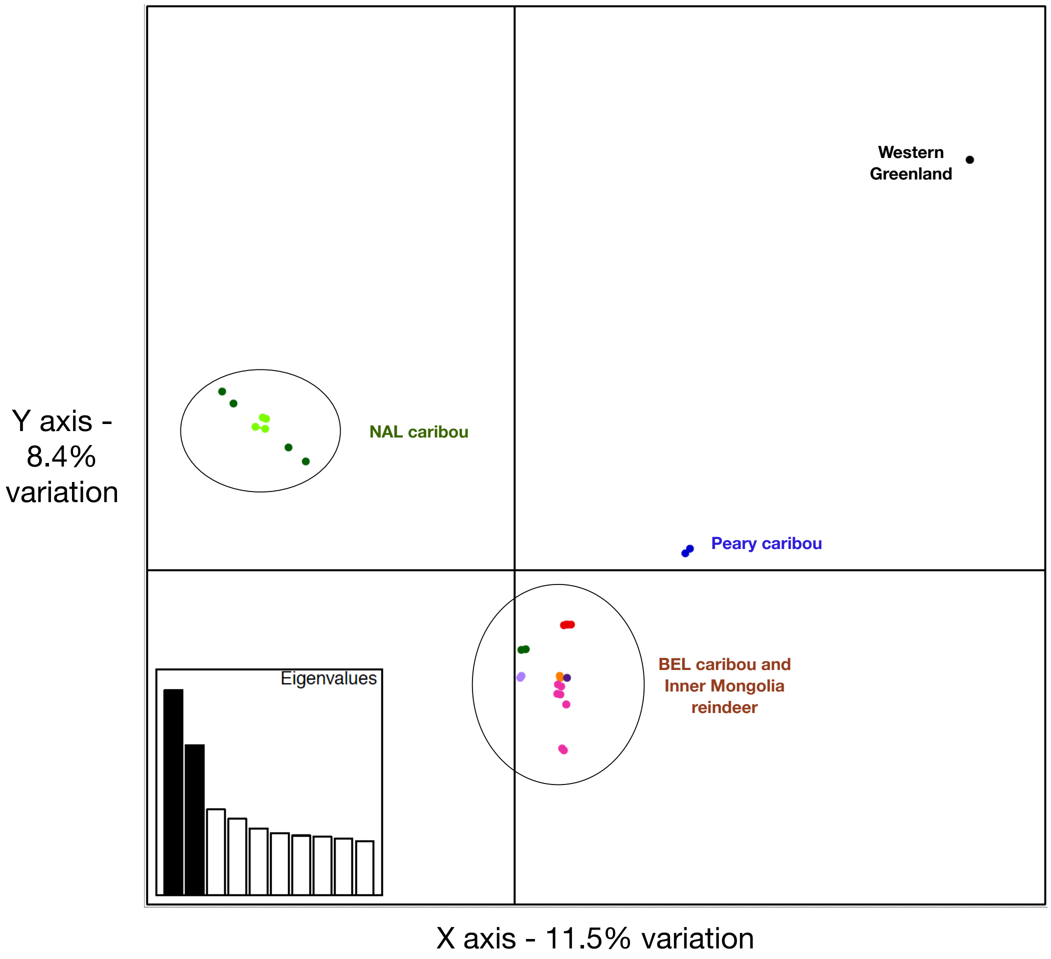

Figure S2 Principal component analysis of 30 caribou and the Inner Mongolia reindeer using an MAF filter removing sites with a frequency less than 0.05. The PCA shows the same patterns as when not using the MAF with the BEL and NAL groups aside from Peary and Greenland which separate out.

MOLECULAR ECOLOGY

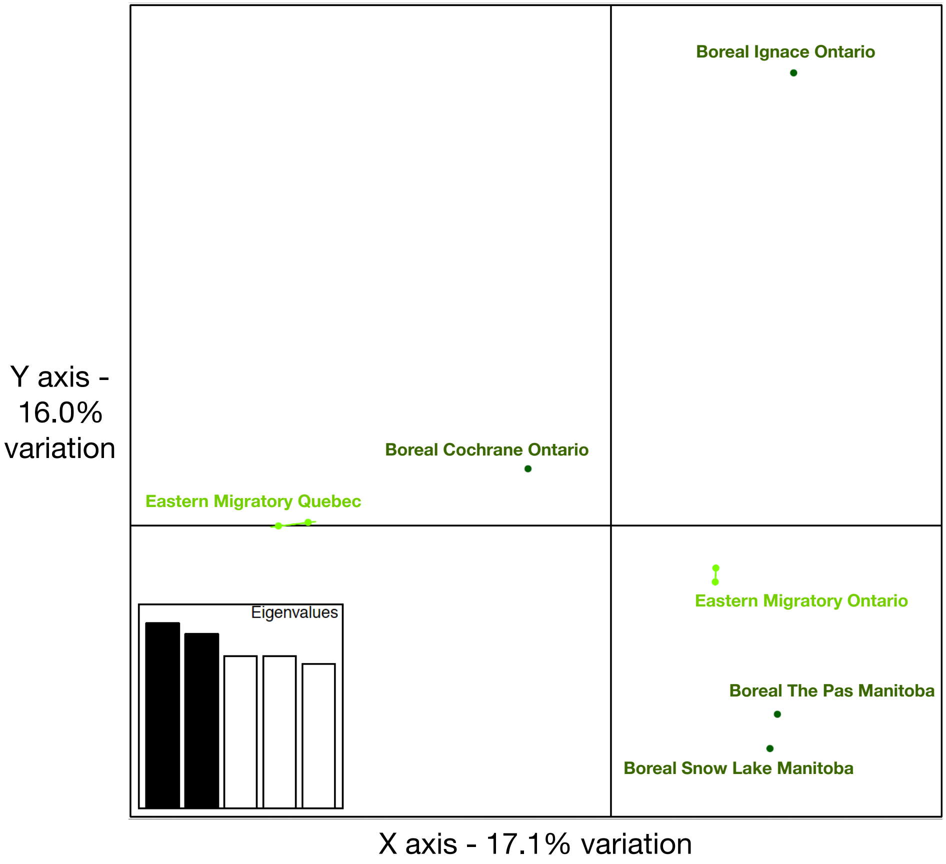

Figure S3 Principal component analysis of NAL caribou using an MAF filter removing sites with a frequency less than 0.05. The PCA shows the same patterns as when not using the MAF with Ignace separating, and eastern migratory caribou from Ontario sitting closest to boreal caribou from Manitoba and those from Quebec sitting closest to boreal caribou from Cochrane.

MOLECULAR ECOLOGY

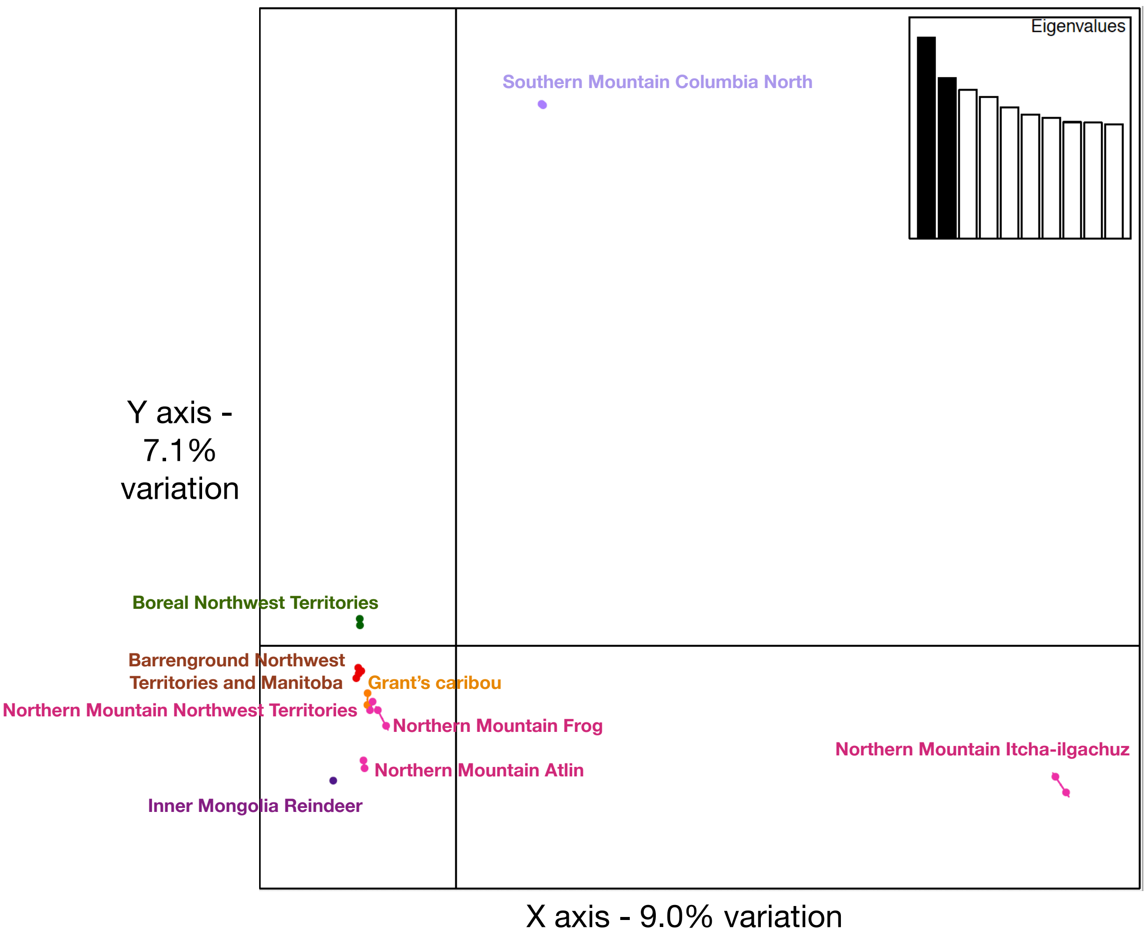

Figure S4 Principal component analysis of BEL caribou excluding Peary and Greenland using an MAF filter removing sites with a frequency less than 0.05. The PCA shows the same patterns as when not using the MAF with southern mountain and northern mountain caribou from Itcha-ilgachuz separating. The inner Mongolia reindeer sits closer to the rest than when not using the MAF, likely due to its higher level of missing data.

MOLECULAR ECOLOGY

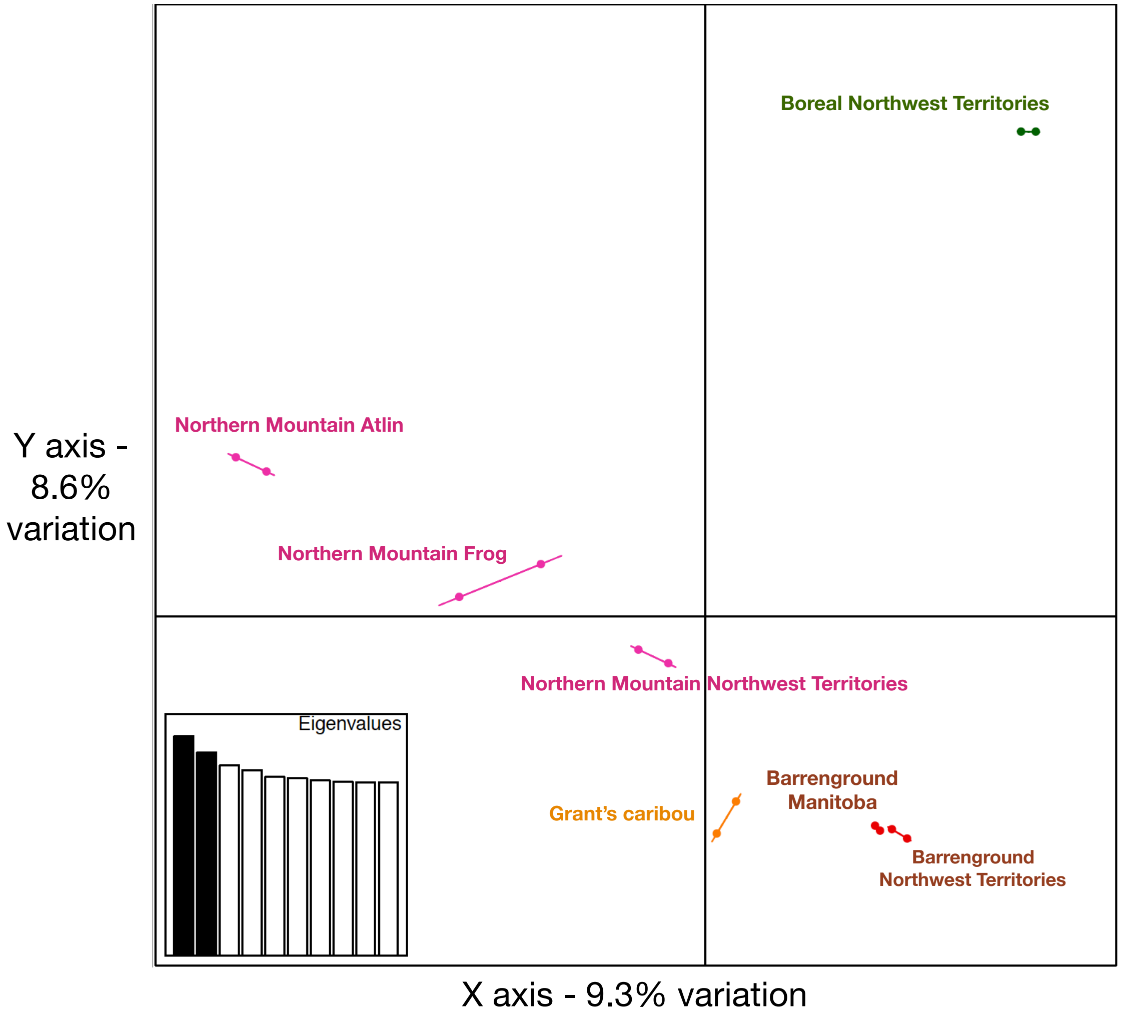

Figure S5 Principal component analysis of a subset of 14 BEL caribou using an MAF filter removing sites with a frequency less than 0.05. The PCA shows the same patterns as when not using the MAF with boreal caribou from the Northwest Territories separating from the others. All groups separate out aside from barrenground caribou from the Northwest Territories and Manitoba which still cluster closely.

MOLECULAR ECOLOGY

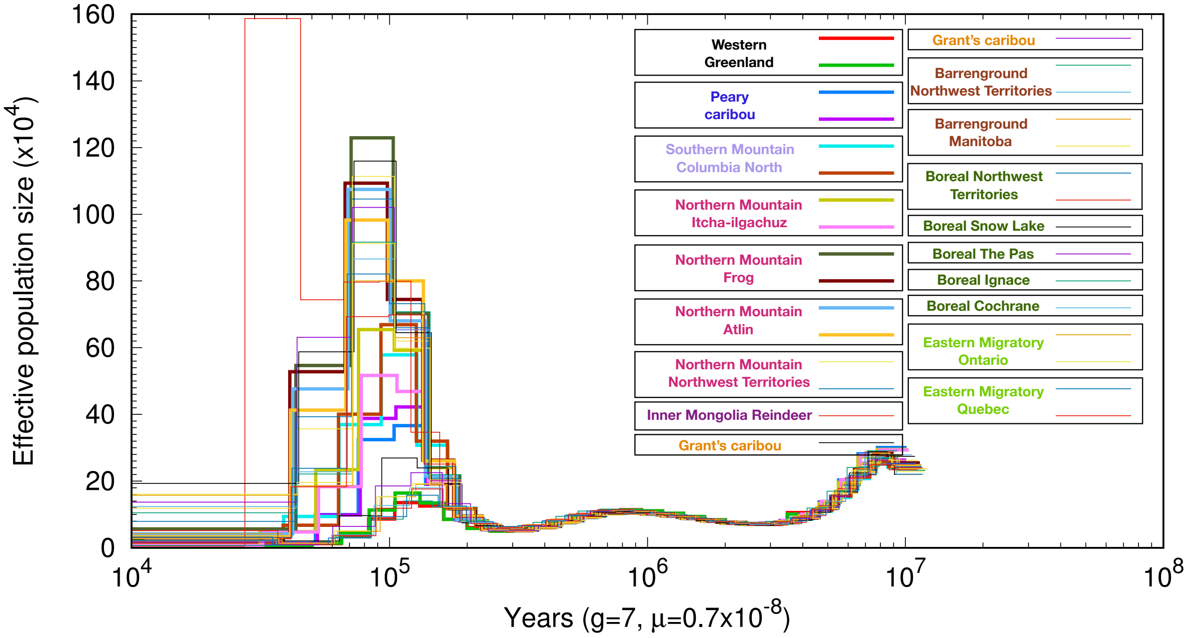

Figure S6 Pairwise Sequentially Markovian Coalescent (PSMC) analysis reconstructing effective population size chances of all 30 caribou and the Inner Mongolia reindeer.

MOLECULAR ECOLOGY

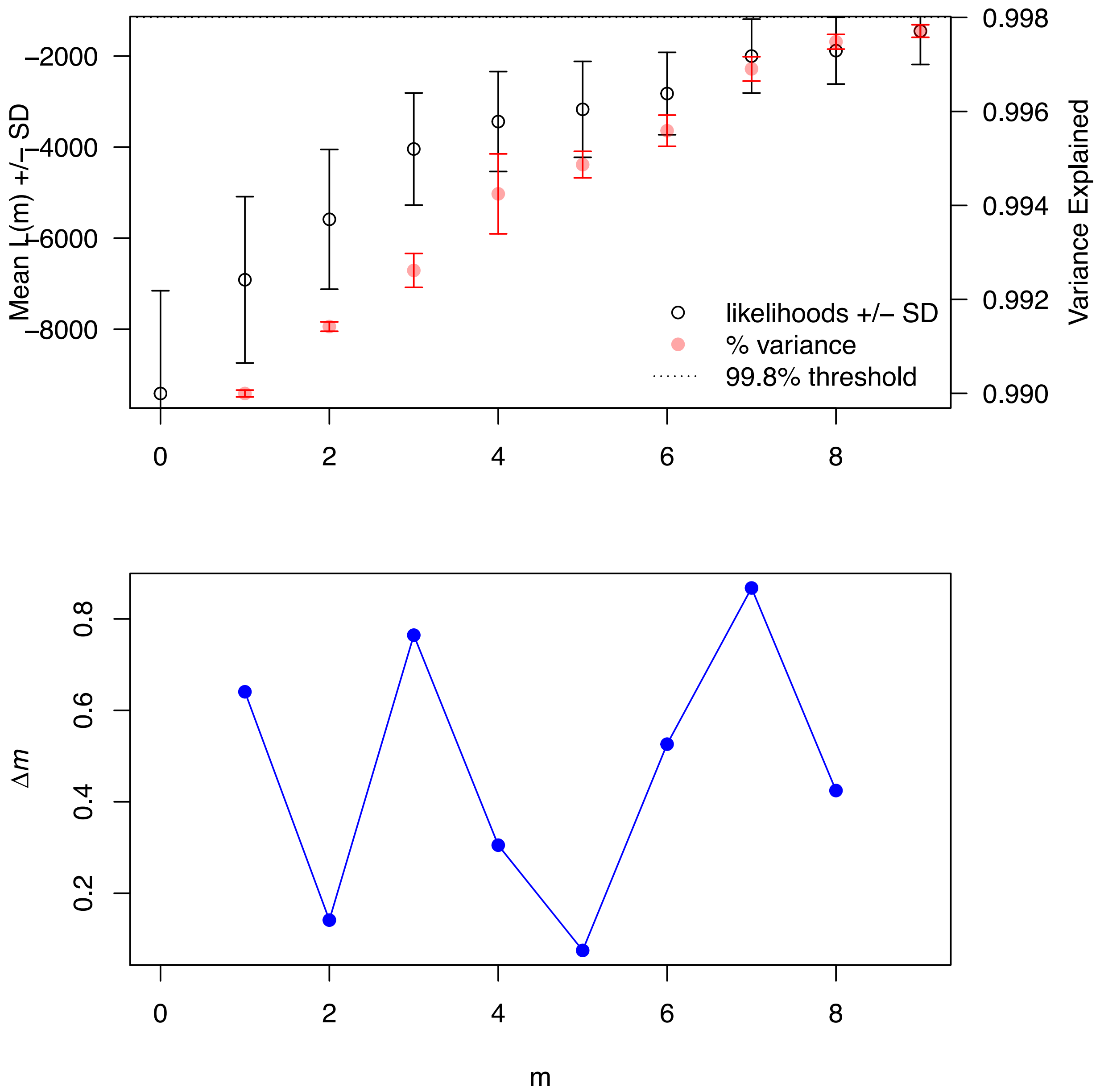

Figure S7 OptM results for Treemix when run for 0-9 migration events. The top plot shows the likelihood (white) and the variance explained (red) of each number of migration events. The bottom plot shows deltaM, which shows the highest peak for 7 migration events. There are also peaks for 1 and 3 migration events, though we show results below for 7 migration events as this includes those inferred for 1 and 3 and because it has the lowest standard error of the three.

MOLECULAR ECOLOGY

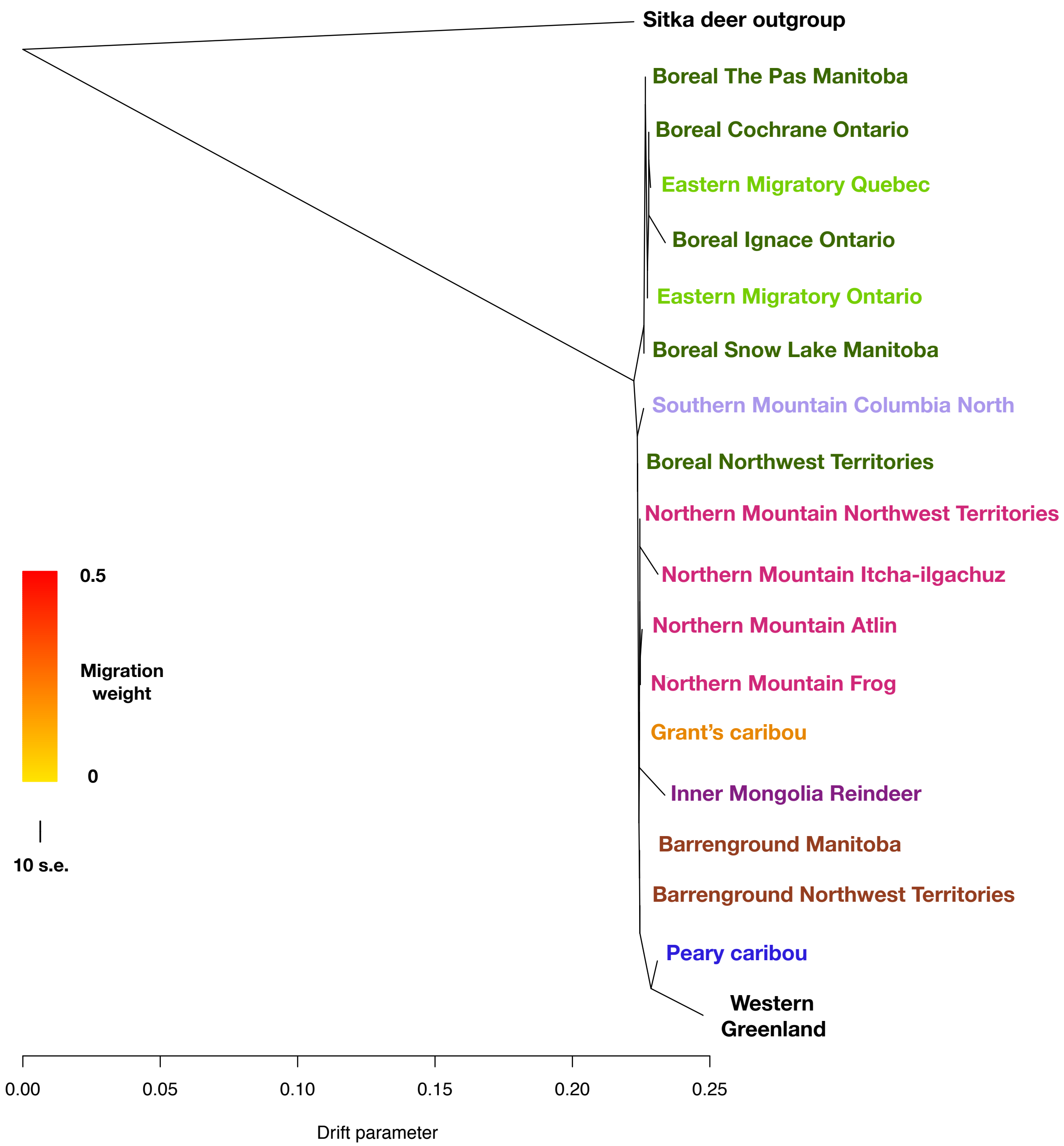

Figure S8 Rooted maximum likelihood phylogeny reconstructed in Treemix with no migration events added. The reconstruction agrees with the BUSCO tree in placing the root splitting the BEL and NAL.

MOLECULAR ECOLOGY

MOLECULAR ECOLOGY

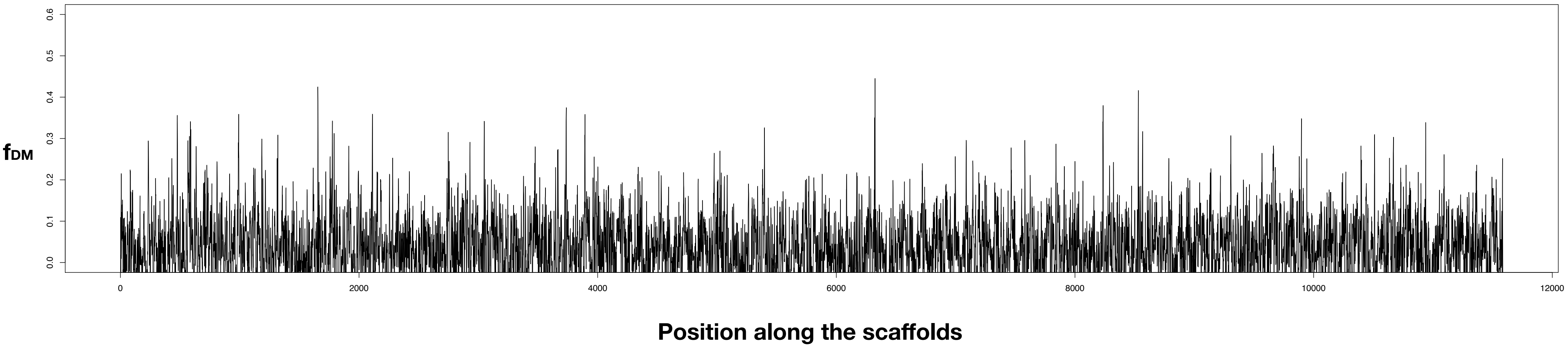

Figure S9 Dsuit plot showing introgressed regions from NAL boreal (Ignace) into Northwest Territories boreal caribou, with Grant's caribou as the sister group. The plot shows the scaffolds in numerical order but the exact order in the genome is not known. Introgressed regions are found throughout the genome on numerous scaffolds (our largest 289 scaffolds represent 90% of the genome assembly and so introgressed regions are likely spread on multiple chromosomes). See supplementary spreadsheet 'Dinvestigate' for the raw data in full.

MOLECULAR ECOLOGY

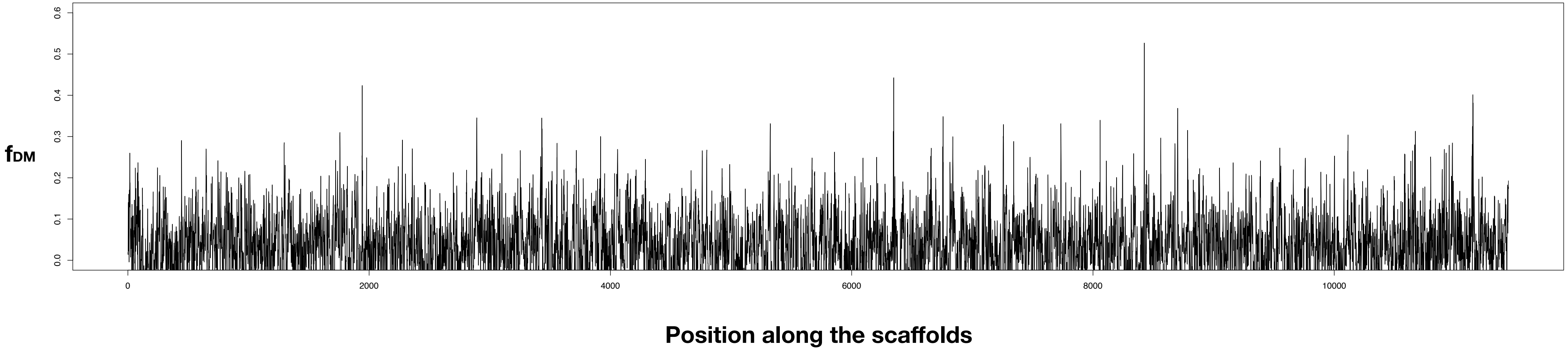

Figure S10 Dsuit plot showing introgressed regions from NAL boreal (Ignace) into southern mountain Columbia North caribou, with Grant's caribou as the sister group. The plot shows the scaffolds in numerical order but the exact order in the genome is not known. Introgressed regions are found throughout the genome on numerous scaffolds (our largest 289 scaffolds represent 90% of the genome assembly and so introgressed regions are likely spread on multiple chromosomes). See supplementary spreadsheet 'Dinvestigate' for the raw data in full.

MOLECULAR ECOLOGY

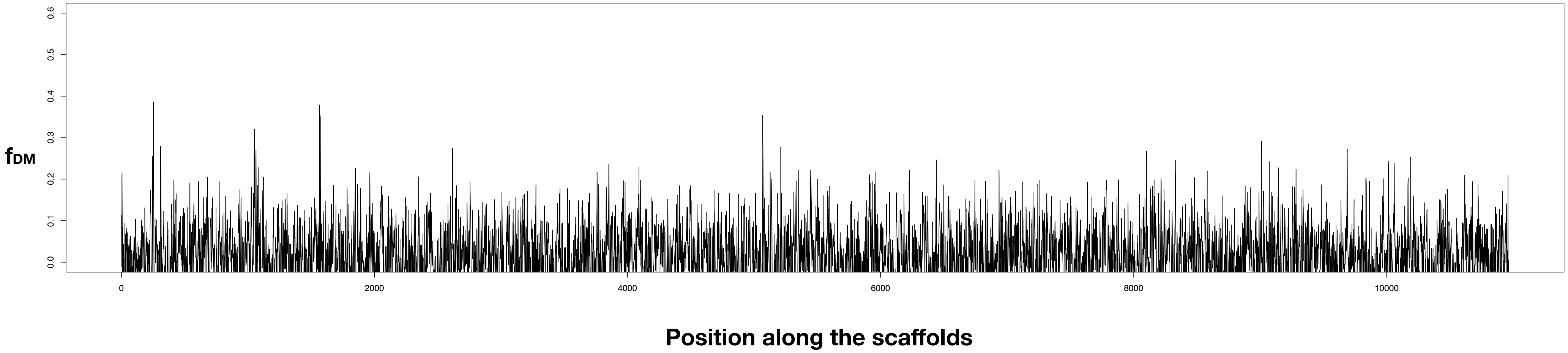

Figure S11 Dsuit plot showing introgressed regions from NAL boreal (Ignace) into northern mountain Itcha-Ilgachuz caribou, with Grant's caribou as the sister group. The plot shows the scaffolds in numerical order but the exact order in the genome is not known. Introgressed regions are found throughout the genome on numerous scaffolds (our largest 289 scaffolds represent 90% of the genome assembly and so introgressed regions are likely spread on multiple chromosomes). See supplementary spreadsheet 'Dinvestigate' for the raw data in full.

MOLECULAR ECOLOGY

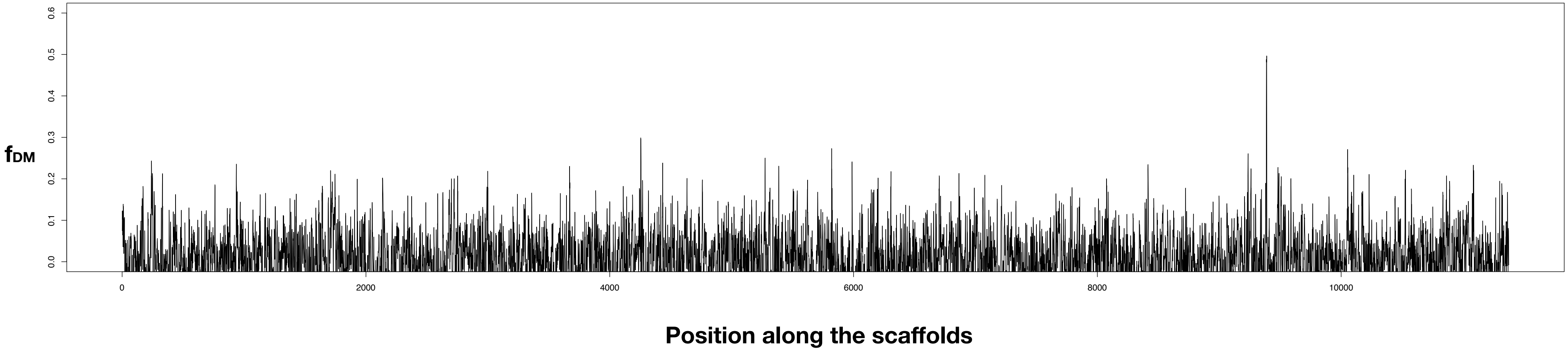

Figure S12 Dsuit plot showing introgressed regions from NAL boreal (Ignace) into northern mountain Frog caribou, with Grant's caribou as the sister group. The plot shows the scaffolds in numerical order but the exact order in the genome is not known. Introgressed regions are found throughout the genome on numerous scaffolds (our largest 289 scaffolds represent 90% of the genome assembly and so introgressed regions are likely spread on multiple chromosomes). See supplementary spreadsheet 'Dinvestigate' for the raw data in full.

MOLECULAR ECOLOGY

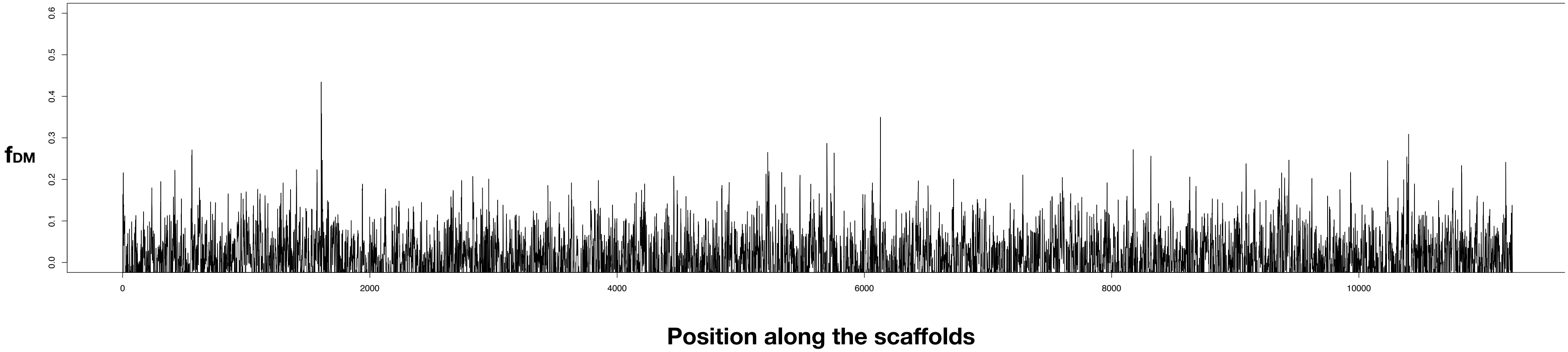

Figure S13 Dsuit plot showing introgressed regions from NAL boreal (Ignace) into northern mountain Atlin caribou, with Grant's caribou as the sister group. The plot shows the scaffolds in numerical order but the exact order in the genome is not known. Introgressed regions are found throughout the genome on numerous scaffolds (our largest 289 scaffolds represent 90% of the genome assembly and so introgressed regions are likely spread on multiple chromosomes). See supplementary spreadsheet 'Dinvestigate' for the raw data in full.

MOLECULAR ECOLOGY

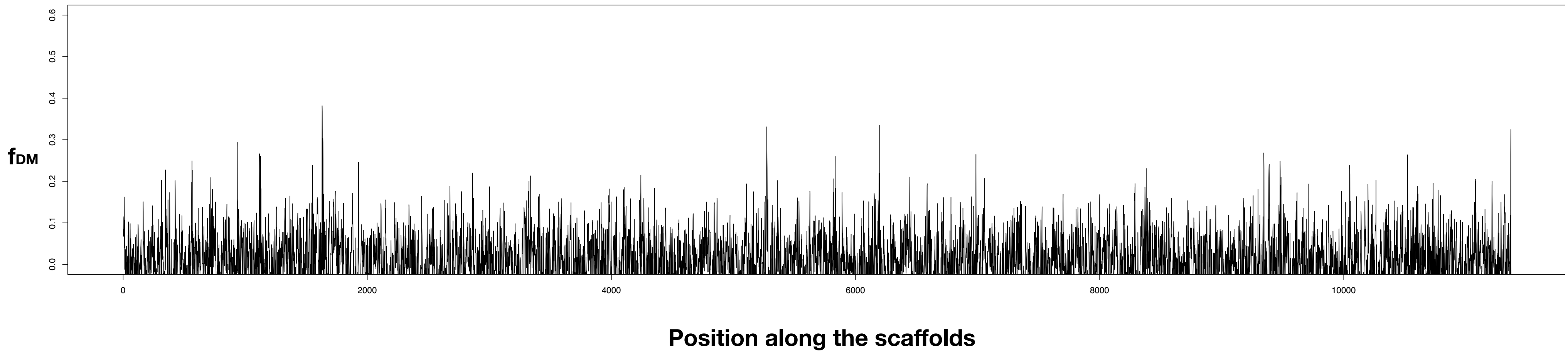

Figure S14 Dsuit plot showing introgressed regions from NAL boreal (Ignace) into northern mountain Northwest Territories Redstone caribou, with Grant's caribou as the sister group. The plot shows the scaffolds in numerical order but the exact order in the genome is not known. Introgressed regions are found throughout the genome on numerous scaffolds (our largest 289 scaffolds represent 90% of the genome assembly and so introgressed regions are likely spread on multiple chromosomes). See supplementary spreadsheet 'Dinvestigate' for the raw data in full.
